# Pirfenidone-Loaded Polymeric Micelles as an Effective Mechanotherapeutic to Potentiate Immunotherapy in Mouse Tumor Models

**DOI:** 10.1021/acsnano.3c03305

**Published:** 2023-12-06

**Authors:** Fotios Mpekris, Petri Ch. Papaphilippou, Myrofora Panagi, Chrysovalantis Voutouri, Christina Michael, Antonia Charalambous, Mariyan Marinov Dinev, Anna Katsioloudi, Marianna Prokopi-Demetriades, Andreas Anayiotos, Horacio Cabral, Theodora Krasia-Christoforou, Triantafyllos Stylianopoulos

**Affiliations:** † Cancer Biophysics Laboratory, Department of Mechanical and Manufacturing Engineering, University of Cyprus, 1678 Nicosia, Cyprus; ‡ Polymers and Polymer Processing Laboratories, Department of Mechanical and Manufacturing Engineering, University of Cyprus, 1678 Nicosia, Cyprus; § Theramir Ltd, R&D Laboratory, 4101 Limassol, Cyprus; ∥ Biomechanics and Living Systems Analysis Laboratory, Cyprus University of Technology, 3036 Limassol, Cyprus; ⊥ Department of Bioengineering, Graduate School of Engineering, The University of Tokyo, Bunkyo, 113-8656 Tokyo, Japan

**Keywords:** nanocarriers, tumor microenvironment, immune checkpoint inhibition, stroma normalization, drug delivery, nano-immunotherapy, oncology

## Abstract

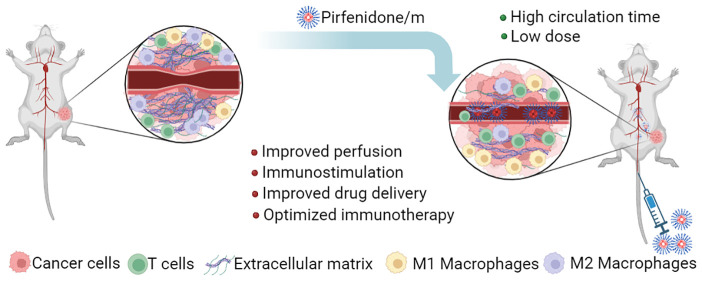

Ongoing research is actively exploring the use of immune checkpoint inhibitors to treat solid tumors by inhibiting the PD-1/PD-L1 axis and reactivating the function of cytotoxic T effector cells. Many types of solid tumors, however, are characterized by a dense and stiff stroma and are difficult to treat. Mechanotherapeutics have formed a recent class of drugs that aim to restore biomechanical abnormalities of the tumor microenvironment, related to increased stiffness and hypo-perfusion. Here, we have developed a polymeric formulation containing pirfenidone, which has been successful in restoring the tumor microenvironment in breast tumors and sarcomas. We found that the micellar formulation can induce similar mechanotherapeutic effects to mouse models of 4T1 and E0771 triple negative breast tumors and MCA205 fibrosarcoma tumors but with a dose 100-fold lower than that of the free pirfenidone. Importantly, a combination of pirfenidone-loaded micelles with immune checkpoint inhibition significantly delayed primary tumor growth, leading to a significant improvement in overall survival and in a complete cure for the E0771 tumor model. Furthermore, the combination treatment increased CD4^+^ and CD8^+^ T cell infiltration and suppressed myeloid-derived suppressor cells, creating favorable immunostimulatory conditions, which led to immunological memory. Ultrasound shear wave elastography (SWE) was able to monitor changes in tumor stiffness during treatment, suggesting optimal treatment conditions. Micellar encapsulation is a promising strategy for mechanotherapeutics, and imaging methods, such as SWE, can assist their clinical translation.

## Introduction

Mechanotherapeutics have been recently introduced as a class of drugs that aim to reprogram components of the tumor microenvironment (TME) in order to reduce/normalize tissue stiffness, improve perfusion and immunostimulation, and thus, enhance efficacy of cytotoxic therapies.^1^ Losartan, a common antihypertensive and angiopoietin receptor blocker, has been a successful example of mechanotherapeutics that effectively improved treatment of patients with locally advanced pancreatic cancer.^2^ Ongoing clinical trials for the use of losartan in combination with chemoradiation and/or immune checkpoint inhibition (ICI) in pancreatic and breast cancer highlight the promise of mechanotherapeutics to be widely added to the treatment regimen of desmoplastic, hard to treat, and thus fatal tumor types (Clinicaltrials.gov identifiers: NCT03563248, NCT05097248). Besides losartan, other mechanotherapeutics entering clinical trials include the endothelin receptor blocker and common antihypertensive bosentan for pancreatic cancer (Clinicaltrials.gov identifier NCT04158635) and the antihistamine ketotifen for sarcomas (EudraCT Number: 2022-002311-39).

The rationale for using mechanotherapeutics to boost cancer therapies is based on the fact that many tumors stiffen as they grow within the host tissue. Tumor stiffening—the sole mechanical aspect of a tumor that patients and clinicians can feel/sense, coupled with the rapid growth of the tumor within the restricted space of the host tissue cause the accumulation of mechanical forces within the tumor. This, in turn, leads to the compression of tumor blood vessels, resulting in hypo-perfusion.^3−6^ Hypo-perfusion and the resulting hypoxia can impose detrimental barriers to the efficacy of therapeutics, including a limited delivery of drugs, immunosuppression, immune exclusion, and an increased metastatic phenotype.^7−11^ Therefore, strategies to normalize the abnormalities in stiffness and mechanical forces in order to improve perfusion and oxygenation have been developed.^12,13^ Mechanotherapeutics that have been tested for this purpose are mainly approved drugs repurposed to normalize the TME. Such drugs include the antihypertensives losartan and bosentan, the corticosteroid dexamethasone, the antihistamines tranilast and ketotifen, and the antifibrotic pirfenidone.^14−19^ The administration of these drugs at a proper dose can enhance tumor perfusion, increase the delivery of chemotherapy, immunotherapy, and nanomedicines, and induce antitumor immunity in preclinical tumor models characterized by abundant compressed vessels.^20−22^

Even though these mechanotherapeutic agents are approved and thus safe for clinical use, they are still subject to dosage limitations and patient exclusion. For instance, in the losartan clinical study,^2^ patients with hypotension were excluded and the losartan dose could not be raised because of its potent antihypertensive effects. Incorporation of mechanotherapeutics into nanoparticle formulations could drastically reduce the dose administered due to enhanced pharmacokinetic characteristics and selective accumulation within the tumor. Indeed, angiotensin receptor blockers and tranilast loaded in nanoparticle formulations have shown to normalize the TME and enhance immunotherapy and nanotherapy at significantly lower concentrations than the free drug.^23−25^ To this end, taking advantage of the mechanotherapeutic properties of pirfenidone,^19^ we developed pirfenidone-loaded polymeric micelles (pirfenidone/m) and explored their potential to facilitate a more efficient normalization of the TME compared to the free drug and at a significantly lower dosage as well as to improve immunostimulation and the efficacy of immune checkpoint inhibition in murine tumor models.

## Results/Discussion

### Characterization of Pirfenidone/m Nanocarriers

**Figure 1:**
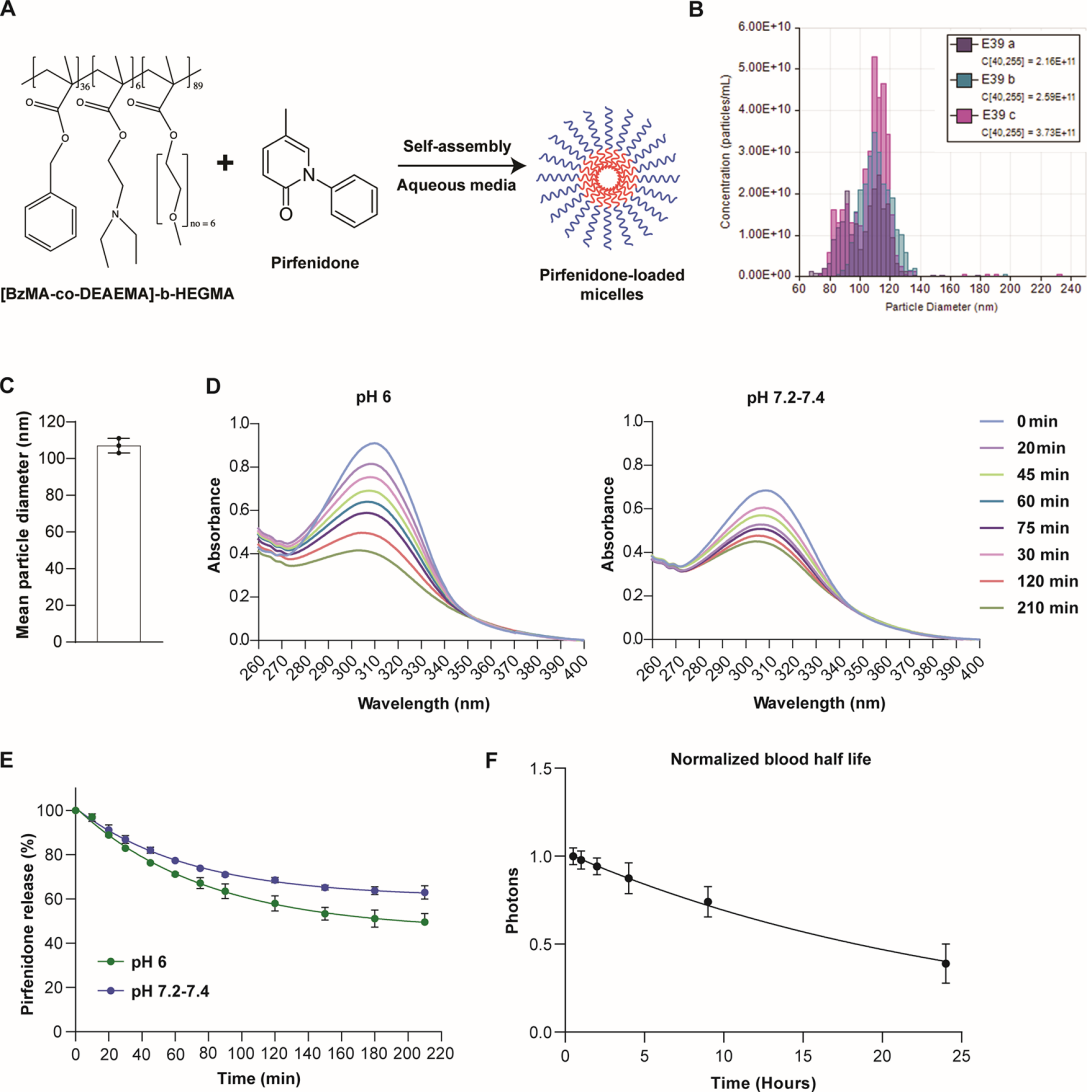
Characterization of pirfenidone/m. (A) Schematic of the process employed for the formation of pirfenidone-loaded micelles in aqueous media. The micelles were generated by mixing [BzMA-*co*-DEAEMA]-*b*-HEGMA and pirfenidone in aqueous conditions. (B) Size distribution of different batches of free micelles and (C) mean diameter size as determined by tunable resistive pulse sensing (TRPS) (*n* = 3). Data presented as the mean ± SE. (D) UV–vis spectra of the pirfenidone-loaded [BzMA_36_-*co*-DEAEMA_6_]-*b*-HEGMA_89_ micelles recorded at different time intervals after being immersed in PBS solution (pH 7.2–7.4) (right plot) and in an aqueous solution at pH = 6.0 (left plot). (E) Pirfenidone release kinetics recorded at different pHs at room temperature: pH 7.2–7.4 (blue), pH 6.0 (green). Average values were recorded from two repetitions. (F) Time-dependent decay of blood concentration after intravenous injection of 10 mg/kg pirfenidone/m i.v. Values are normalized with the data from the first time point, i.e., 30 min. Data are shown as the mean ± SE (*n* = 4 mice).

Amphiphilic micellar drug nanocarriers were synthesized, consisting of a HEGMA water-soluble corona and a water-insoluble core bearing aromatic BzMA moieties combined with pH-responsive, cationic DEAEMA units in a random copolymer structure, and were prepared and further employed as nanocontainers for the encapsulation of pirfenidone. On one hand, the HEGMA polymer segments constituting the hydrophilic micellar corona (shown in blue color in Figure 1A) exhibit high biocompatibility and improved blood circulation times.^26^ On the other hand, the incorporation of the benzyl moieties as well as of the tertiary amino functionalities in the BzMA-*co*-PDEAEMA random copolymer chains constructing the micellar core (shown in red color in Figure 1A) aimed at (i) enhancing the drug loading efficiency via the development of π–π attractive forces developed among the benzene aromatic rings of the BzMA units and of pirfenidone and (ii) promoting the pH-responsive release of pirfenidone under acidic environments, since DEAEMA functionalities exhibiting a p*K*_a_ = 7.3 turn from neutral/hydrophobic into cationic/hydrophilic by lowering the solution pH.^27^ Tunable resistive pulse sensing (TRPS) technology was employed to determine the concentration and size distribution of the micellar nanoparticles. Specifically, the mean micellar diameter was determined to be 107 nm following analysis of three different batches (Figure 1B,C). The pH-responsive release of pirfenidone was recorded at different time intervals upon immersing the micellar solution-containing dialysis cassette in PBS (pH 7.2–7.4) and in an aqueous solution of lower pH (pH = 6) (Figure 1D). As expected, under physiological pH conditions, the absorption signal appearing at 310 nm corresponding to pirfenidone that is encapsulated within the micelles decreases slowly, reaching a 35% release percentage after 3 h. Likewise, by performing the drug release study at a lower pH (pH = 6) the absorption signal decreased in a slightly but statistically significant faster rate (*p* = 0.0011 for 210 min). This is due to the existence of a small number (i.e., 6 units) of the pH-responsive DEAEMA moieties within the copolymer, which increases the drug release rate to some extent when the pirfenidone-loaded micelles are exposed in acidic conditions, reaching a 51% release percentage after 3 h (Figure 1E). The micelles also achieved a long circulation time, being detected in the blood of healthy mice for more than 24 h, exhibiting a blood half-life of the β-phase of 27 h (Figure 1F). We further studied the distribution of the micelles (labeled with the fluorophore DiR) in mice bearing 4T1 breast tumors at 24 and 48 h postadministration and found that their intratumoral accumulation remains the same (Supplementary Figure S1). Finally, the impact of exposure to pirfenidone/m on overall cell viability of 4T1 breast cancer cells was monitored through the quantification of mitochondrial activity using the MTT assay. The viability of pirfenidone/m compared to the micelles without the pirfenidone load was tested at the concentration of 0.1 μg/mL following 6, 24, and 48 h of incubation, demonstrating a noncytotoxic effect (Supplementary Figure S2).

### Pirfenidone/m Effectively Normalize the TME in a 100-fold Reduced Dose

**Figure 2:**
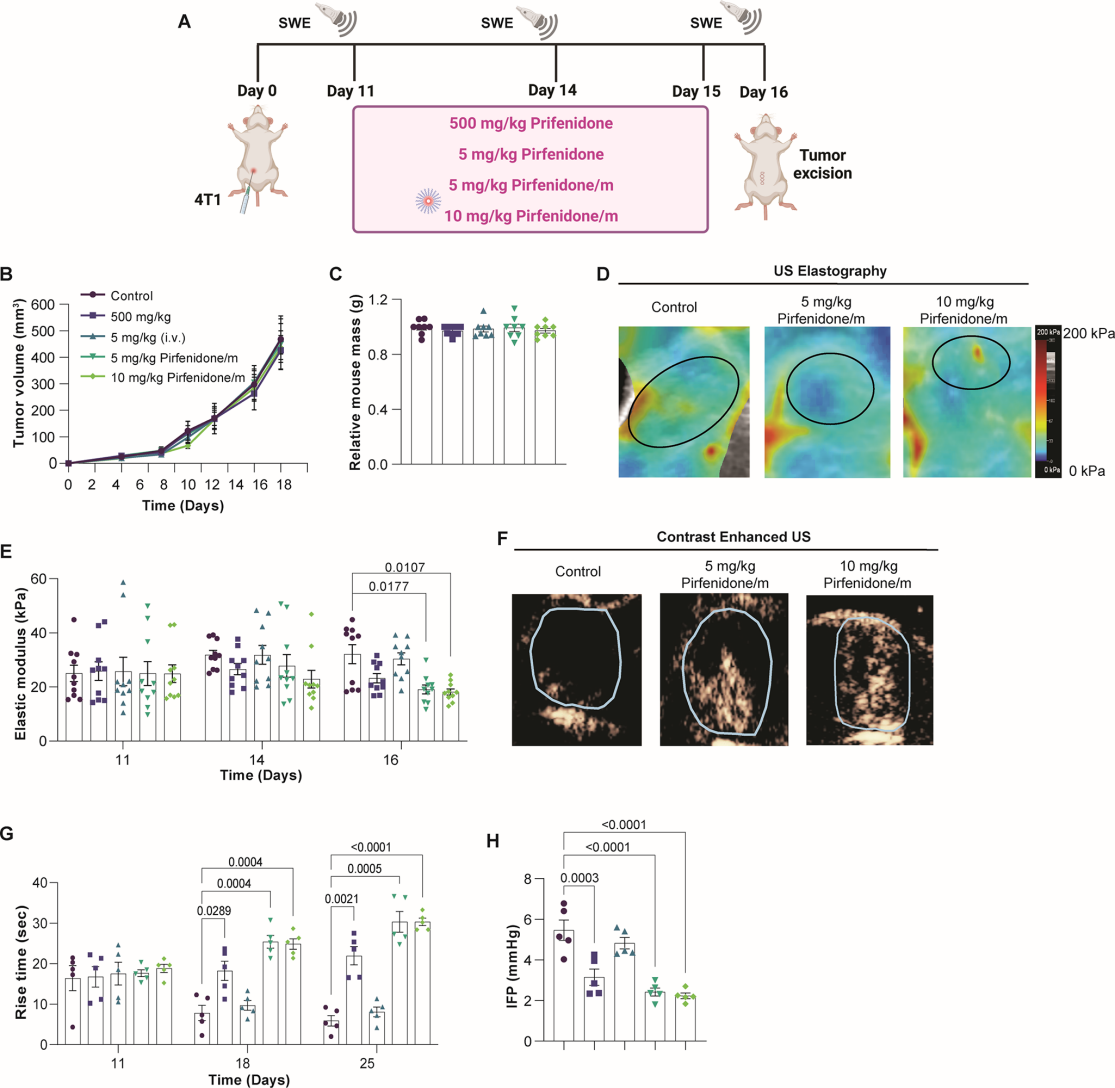
Pirfenidone/m modulate the physical TME. (A) Schematic of the experimental protocol created with BioRender.com. Effects of free pirfenidone and pirfenidone/m on 4T1 tumor growth (B) (*n* = 5 mice) and (C) mouse mass (*n* = 5 mice). (D) Representative SWE images for the control group and the two groups receiving pirfenidone/m at the end of the experimental protocol. The black line denotes the tumor margin. (E) Elastic modulus values measured with SWE (*n* = 5 mice, *N* = 2 image fields per mouse). (F) Representative images depicting the spatial distribution of microbubbles with contrast enhanced ultrasound (CEUS) at the time of peak intensity obtained at the end of the experimental protocol (*n* = 5 mice). (G) Rise time measured with CEUS (*n* = 5 mice). (H) Interstitial fluid pressure (IFP) measured with the wick-in-needle technique (*n* = 5 mice). Data are presented as mean ± SE. Statistical analyses were performed using two-way ANOVA with multiple comparisons of the Dunnett test.

We first tested the ability of pirfenidone/m to induce TME normalization when administered in amounts significantly lower than those of the free drug. Mice bearing syngeneic, orthotopic 4T1 breast tumors were treated with either free pirfenidone or pirfenidone/m (Figure 2A). Free pirfenidone was administered daily at the dose of 500 mg/kg orally^19^ or by intravenous injection (i.v.) at the dose of 5 mg/kg. Pirfenidone/m were administered i.v. at the dose of 5 or 10 mg/kg daily, i.e., 100 and 50 times lower than the oral administration of the free drug. No significant effects on tumor growth or the body mass of mice were observed with any of the administered doses (Figure 2B,C). The impact of various pirfenidone treatments on normalizing the TME was examined in vivo, focusing on physical properties such as stiffness, perfusion, and interstitial fluid pressure (IFP). Tumor stiffness and perfusion were measured with ultrasound shear wave elastography (SWE) and contrast enhanced ultrasound (CEUS), respectively, whereas IFP was measured with the wick-in-needle method.^5,15,28−30^ Tumor stiffness was measured as the average elastic modulus of the tumor, and for perfusion, the rise time (RT) was employed, derived from the time–intensity curve created by the transfer of a bolus of microbubbles through the tumor tissue. The groups that received either the 5 or the 10 mg/kg pirfenidone/m had a statistically significant reduction in stiffness and IFP and an increase in perfusion compared to the control group (Figure 2D–H). Also, when treatment with pirfenidone/m is compared to the 500 mg/kg free pirfenidone, we observe that pirfenidone/m can more effectively alleviate stiffness, improve perfusion, and increase IFP, even though in some cases the difference is not statistically significant. Interestingly, the administration of 5 mg/kg free pirfenidone i.v. did not have any effects on the TME, presumably due to its rapid clearance. Furthermore, treatment with pirfenidone/m did not have any impact on tissue stiffness of major healthy organs, such as the liver, spleen, and kidneys (Supplementary Figure S3).

**Figure 3:**
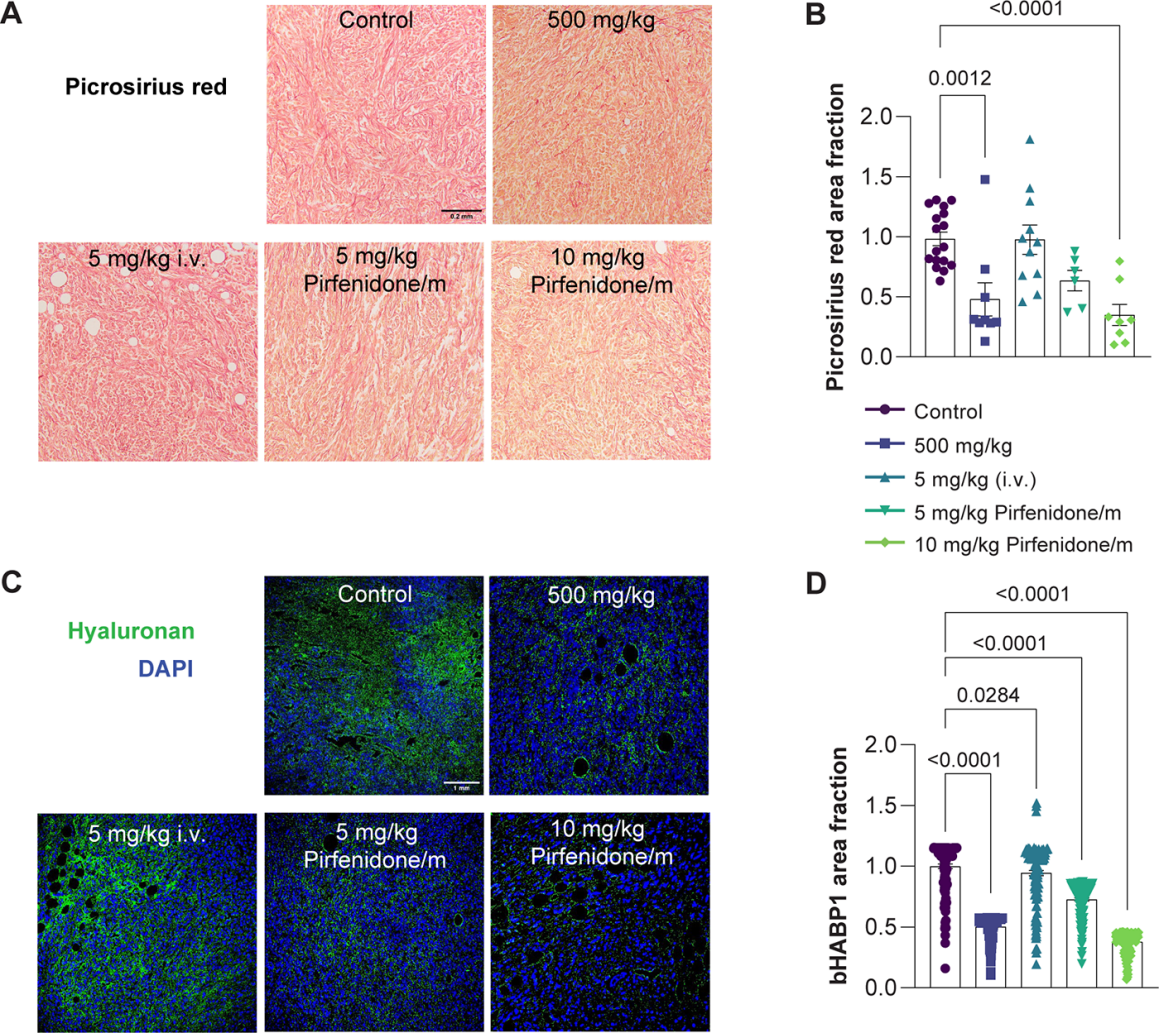
Pirfenidone/m effectively decrease collagen and hyaluronan levels. (A) Representative light microscope images of Picrosirius red staining (red color). Black scale bar indicates 0.2 mm. (B) Graph depicting the area fraction of Picrosirius red, representing collagen fibers in tissue sections, normalized to the average value in control tumors (*n* = 3 mice; *N* = 3 or 4 image fields). (C) Immunofluorescence images showing hyaluronan binding protein staining (bHABP1, green) counterstained with nuclear staining (in blue). The white scale bar denotes a size of 1 mm. (D) Graph of the area fraction of bHABP1 in immunofluorescence images (*n* = 3 mice, *N* = 3 or 4 image fields). Data are presented as mean ± SE. Statistical analyses were performed by using ordinary one-way ANOVA with multiple comparison Dunnett test.

The restoration of physical properties in desmoplastic tumors, as observed in the tumor models examined in our study, is attributed to a large extent to the hyaluronan and collagen content.^7,31,32^ Pirfenidone targets both these components.^19^ We assessed the levels of collagen and hyaluronan using histological analysis and fluorescence immunostaining, respectively. Indeed, we found a decrease in collagen and hyaluronan protein levels following treatment with pirfenidone/m at both doses similar to the free drug (500 mg/kg orally) and which ranged from 40% to 60% compared to the control group (Figure 3). Treatment with pirfenidone/m did not affect collagen and hyaluronan levels of major healthy organs (Supplementary Figure S4). Therefore, pirfenidone/m can effectively normalize the TME, reducing drastically the required dose of free pirfenidone.

### Pirfenidone/m Enhance Drug Delivery and Antitumor Efficacy of Immunotherapy

Subsequently, we examined the capacity of pirfenidone/m to enhance drug delivery and the antitumor effectiveness of immune checkpoint inhibitors (ICIs). BALB/c mice bearing 4T1 tumors were pretreated with either pirfenidone/m (10 mg/kg, i.v.) or control for 6 days and then administered an intravenous injection of DiR-labeled pirfenidone-free micelles of the same composition. Pretreatment with pirfenidone led to an improved and more uniform accumulation of DiR-labeled micelles and fluorescent immunotherapy in the tumor site (Supplementary Figures S5–S7), affirming that TME normalization induced by pirfenidone/m can enhance drug delivery.

**Figure 4:**
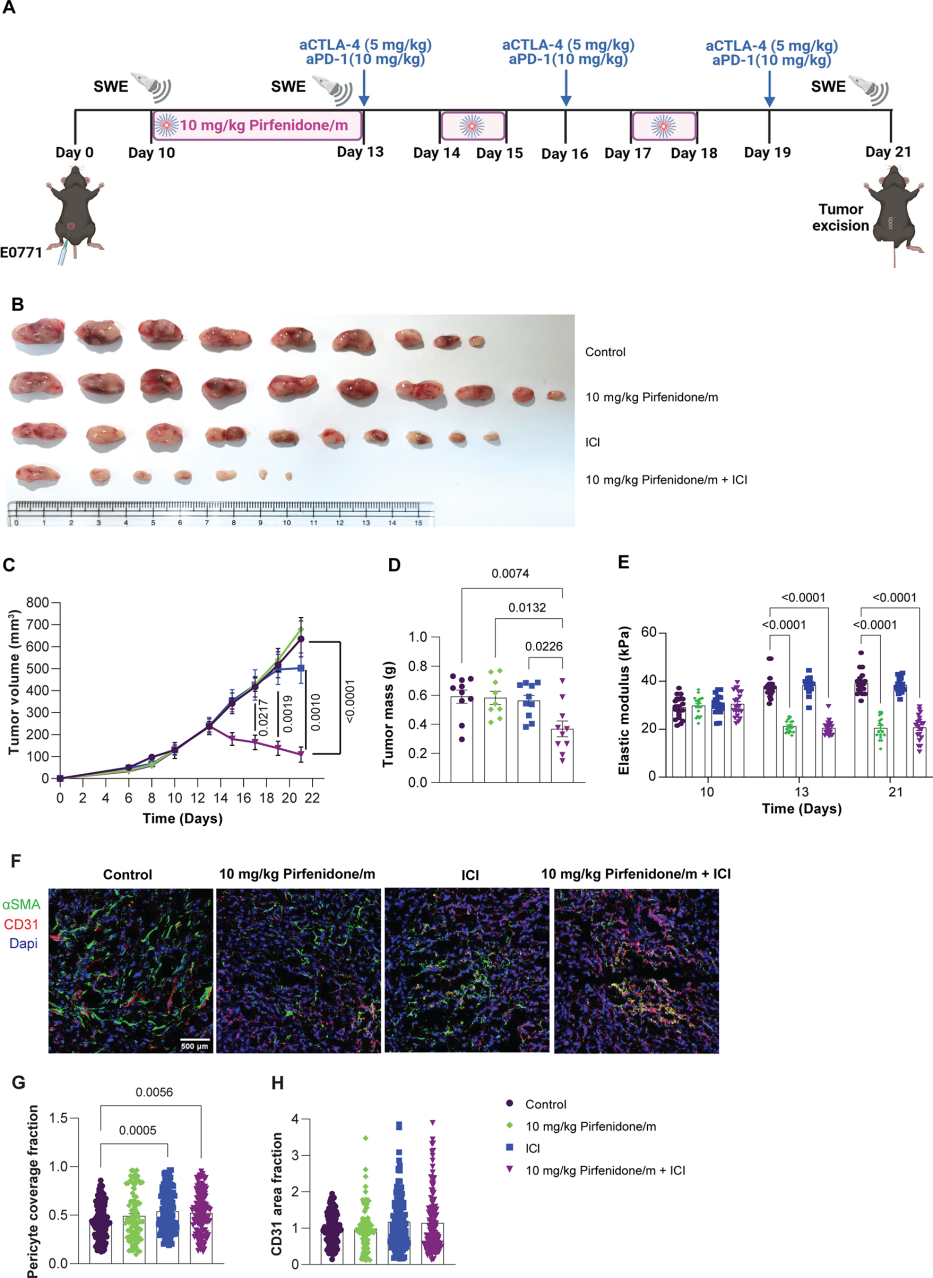
Pirfenidone/m significantly enhances the effectiveness of immunotherapy. (A) Experimental treatment protocol. Created with BioRender.com. (B) Images of E0771 tumors after removal (*n* = 9 or 10 mice, in the group of pirfenidone/m + ICI, three mice were cured). (C) Tumor growth (*n* = 9 or 10 mice), (D) tumor mass (*n* = 9 or 10 mice), and (E) tumor elastic modulus (*n* = 9 or 10 mice, *N* = 2 images field per mouse) measured with SWE of E0771 tumors treated with pirfenidone/m and anti-CTLA4/anti-PD-1 antibodies. (F) Representative immunofluorescence images of nuclear marker (blue), CD31 endothelial marker (red), and αSMA pericyte marker (green) immunostaining of 4T1 breast tumors treated as indicated. White scale bar indicates 0.5 mm. (G) Quantification of pericyte coverage fraction determined by the colocalization of CD31 and αSMA. (H) Quantification of the CD31 area fraction following immunostaining with an anti-CD31 endothelial cell marker. Data are presented as mean ± SE. Statistical analyses were performed by using for (C) mixed-effects analysis with multiple comparisons Tukey test, for (D, G, H) ordinary one-way ANOVA with multiple comparisons Dunnett test, and for (E) two-way ANOVA with multiple comparisons Dunnett test.

Then, we explored the antitumor efficacy of ICIs combined with the pirfenidone/m. For this and subsequent experiments, the 10 mg/kg dose of pirfenidone/m was employed. Initially, we investigated the ability of pirfenidone/m to sensitize the TME to immune checkpoint inhibition by altering PD-L1 levels (Supplementary Figure S8**)**. No significant changes were observed after pirfenidone/m treatment compared to the untreated group. To examine the combined effect of pirfenidone/m with ICIs, animals were treated with control solution (anti-IgG, H_2_O), pirfenidone/m, ICIs cocktail (5 mg/kg anti-CTLA4, 10 mg/kg anti-PD-1), and combination therapy of pirfenidone/m and ICIs (Figure 4A, Supplementary Figure S9A, Supplementary Figure S10A). Immunotherapy alone had no antitumor effects, but treatment with pirfenidone/m potentiated anti-CTLA4/anti-PD-1 therapy in both tumor models in terms of significant reduction in tumor volume and mass (Figure 4B–D, Supplementary Figure S9B–D, Supplementary Figure S10A). Additionally, immunotherapy alone had no effect on tumor elastic properties measured with SWE compared to the control group and did not affect the elastic modulus of the groups treated with pirfenidone/m (Figure 4E, Supplementary Figure S9E, Supplementary Figure S10A). Also, we examined the effect of various treatments on the cancer-associated fibroblast (CAF) population and activity, which could result in a decrease in tumor elastic properties through remodeling of the extracellular matrix (Supplementary Figure S11). The combination of pirfenidone/m and ICIs decreases (not with a statistical significance) the population and activity of CAFs. Lastly, we explored the effect of the combined treatment on the coverage of blood vessels by pericytes (Figure 4F–H), which is an indicator of vascular normalization. In contrast with host tissue, in tumors the absence of pericyte coverage leads to increased vessel permeability and decreased drug delivery. Interestingly, we found that ICI alone or in combination with pirfenidone/m significantly increases the pericyte coverage in 4T1 tumors (Figure 4G) without causing any effect on the overall count of vessels (Figure 4**H**).

### Inclusion of Pirfenidone/m to ICI Treatment Enhances T Cell Infiltration

**Figure 5:**
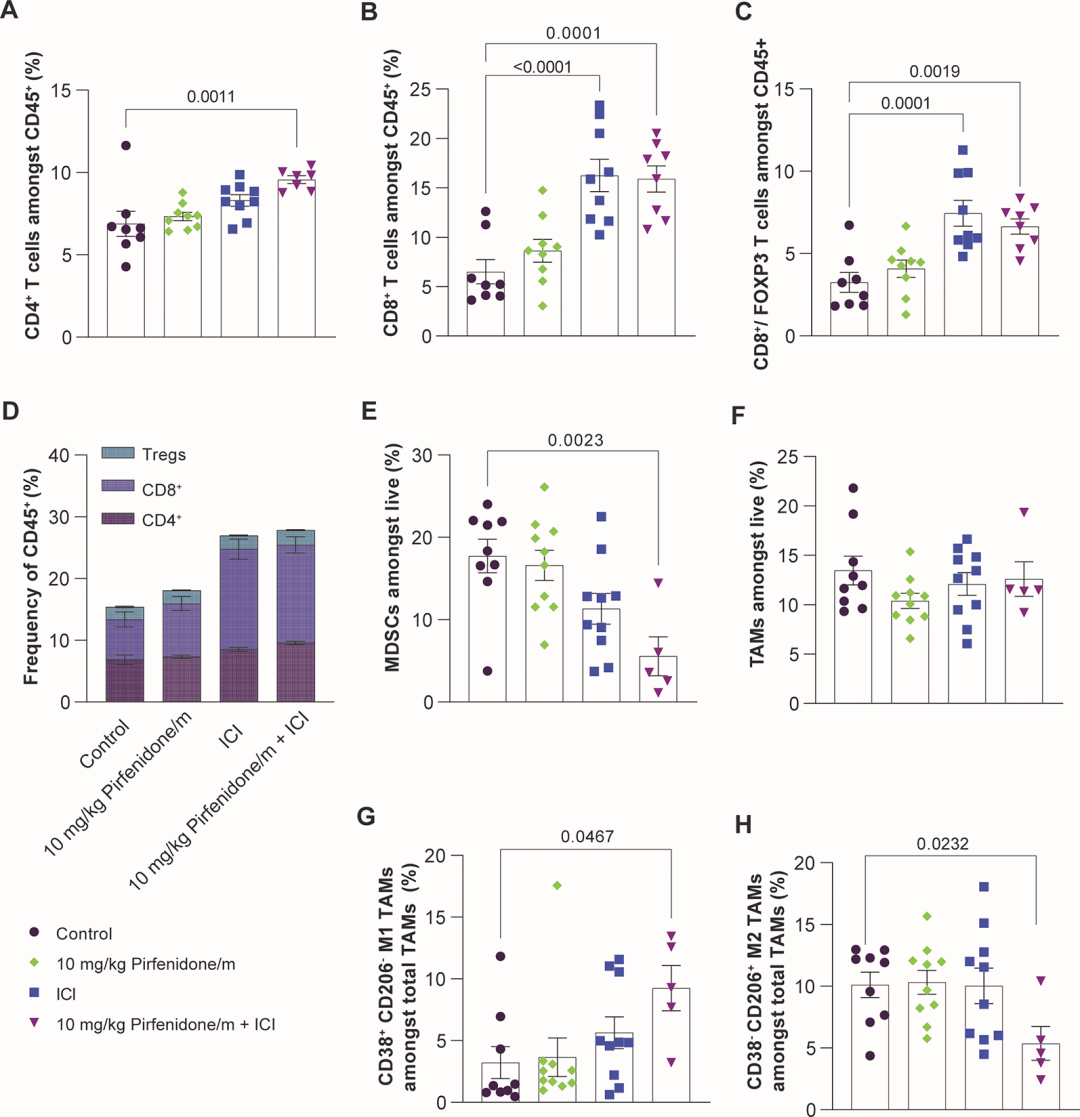
Pirfenidone/m and ICI increase immune cell infiltration and induce immunostimulation. Quantification of (A) CD3^+^ CD4^+^ (SP, single positive) and (B) CD3^+^ CD8^+^ (SP, single positive) cells among CD45^+^ lymphocytes (*n* = 6–10 mice). (C) Ratio of cytotoxic CD8^+^ T cells to immunosuppressive CD4^+^ regulatory T cells (Tregs) (*n* = 6–10 mice). Tregs are defined as Foxp3^+^CD127^lo^CD25^hi^ CD4 SP gated on CD45^+^ lymphocytes. (D) Frequency of CD4^+^, CD8^+^, and Tregs in total lymphocyte population (*n* = 6–10 mice). (E) Quantification of intratumoral MDSCs (CD45^+^ CD11b^+^ GR1^+^) and (F) TAMs (CD45^+^ CD11b^+^ GR1^–^ F4/80^+^) gated on live cells. Percentage of antitumor (G) M1-like TAMs (CD45^+^ CD11b^+^ GR1^–^ F4/80^+^CD206^–^CD38^+^) and (H) M2-like TAMs (CD45^+^ CD11b^+^ GR1^–^ F4/80^+^CD206^+^CD38^–^) gated on total TAM population (*n* = 6–10 mice). Data are presented as mean ± SE. Statistical analyses were performed by using ordinary one-way ANOVA with multiple comparisons Dunnett test.

**Figure 6:**
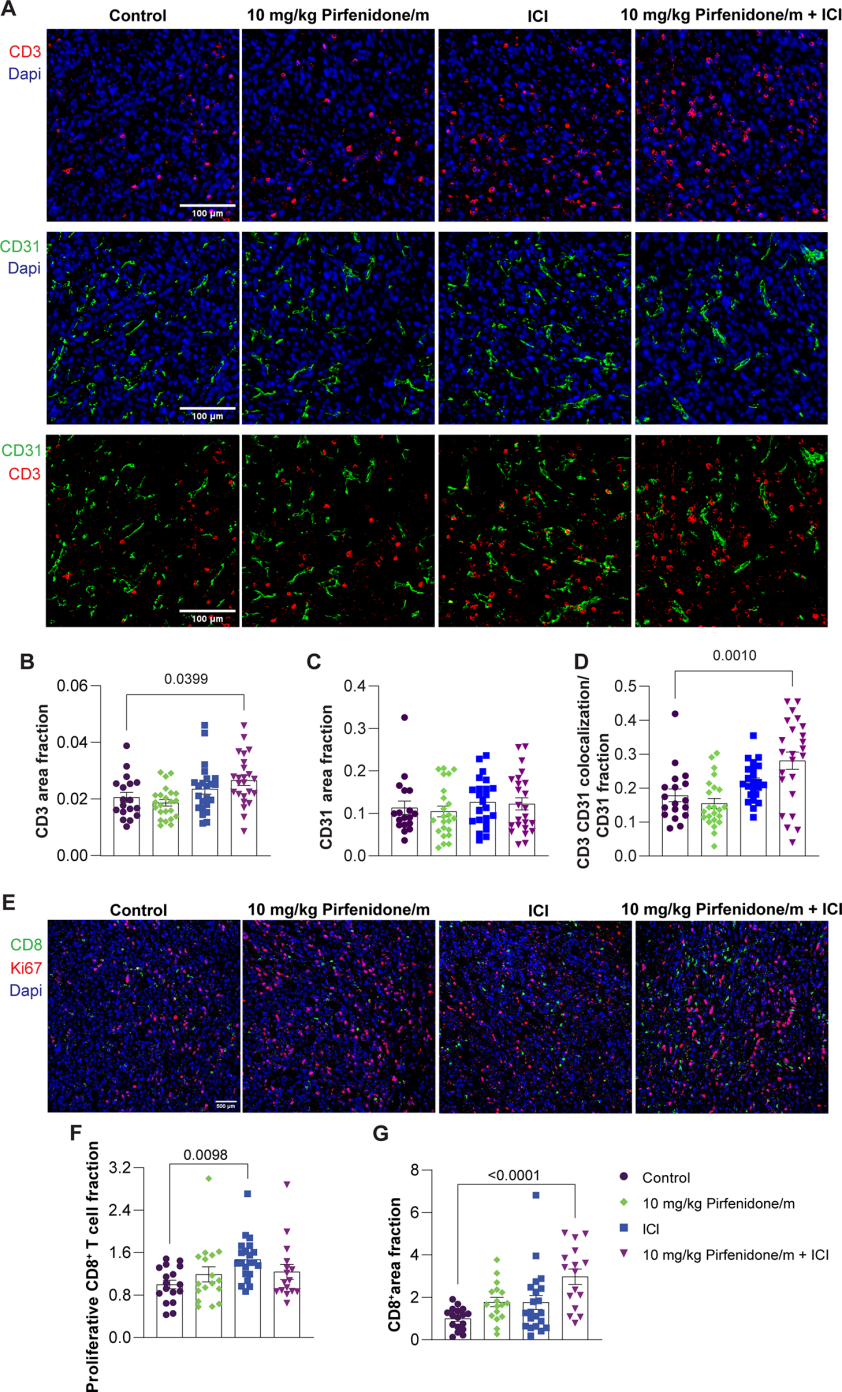
Pirfenidone micelles combined with ICI increase CD3 and CD8^**+**^ T cell density in 4T1 tumors. (A) Representative immunofluorescence images of CD3 staining (red) and CD31 staining (green) counterstained with nuclear staining (blue). White scale bar indicates 0.1 mm. (B) Graph illustrating the area fraction of the CD3 T cell marker in immunofluorescence images standardized to DAPI nuclear staining. (C) Graph depicting the area fraction of the CD31 marker in immunofluorescence images, normalized to DAPI nuclear staining. (D) Graph of the overlapping signal of the CD3 T cell and CD31 endothelial marker in immunofluorescence images. (E) Representative immunofluorescence images of CD8^+^ T cell staining (green) and proliferation marker, Ki67 staining (red), counterstained with DAPI nuclear staining (blue). White scale bar indicates 0.5 mm. (F) Graph illustrating the area fraction of Ki67 colocalized with CD8^+^ relative to the total CD8^+^ area in immunofluorescence images. (G) Graph of the area fraction of the T cell marker CD8^**+**^ in immunofluorescence images normalized to DAPI stain. Data are presented as mean ± SE. Statistical analyses were performed by using ordinary one-way ANOVA with multiple comparisons Dunnett test. *P*-values less than 0.05 are denoted on the graphs.

As tumor perfusion correlates with heightened immune cell infiltration and activity, we aimed to investigate whether the robust antitumor responses observed with pirfenidone/m in combination with ICIs are dependent on the levels of tumor immunogenicity. Flow cytometry analysis revealed that immunotherapy treatment increases the ratio of cytotoxic CD8^+^ T cells to immunosuppressive regulatory T cell (Tregs) (Figure 5A–D, Supplementary Figure S12). We also performed immunofluorescence staining for the pan T cell marker, CD3, in the 4T1 tumors treated with pirfenidone/m, ICI, or their combination and found that only the combination could significantly increase intratumoral T cell levels, particularly at the tumor center (Figure 6A,B, Supplementary Figure S13). Adding to this, we also demonstrated that these T cells accumulate close to blood vessels, as indicated by the overlapping fluorescence signal of the endothelial markers CD31 and CD3 (Figure 6C,D), suggesting that restoring vessel functionality with pirfenidone primes the TME for immunotherapy. Additionally, ICI treatment increased the intratumoral recruitment of proliferative cytotoxic CD8^+^ T cells, whereas the combination of ICI with pirfenidone/m increased CD8^+^ T cell density (Figure 6E–G). Levels of Tregs do not change over any treatment, suggesting that the immunotherapy effect is primarily dependent on the CD8^+^ T cell population (Figure 5D). Furthermore, ICI treatment reduces intratumoral levels of immunosuppressive MDSCs, while the combination with pirfenidone/m enhances this effect (Figure 5E). ICI treatment does not affect overall macrophage levels but reverts their immunosuppressive phenotype as indicated by the reduction in M2-like phenotype (CD38^–^ CD206^+^) and upregulation of antitumor M1-like phenotype (CD38^+^ CD206^–^) (Figure 5F–H).

### Pirfenidone/m and Immunotherapy Combination Enhances Overall Survival and Triggers Immunological Memory

**Figure 7:**
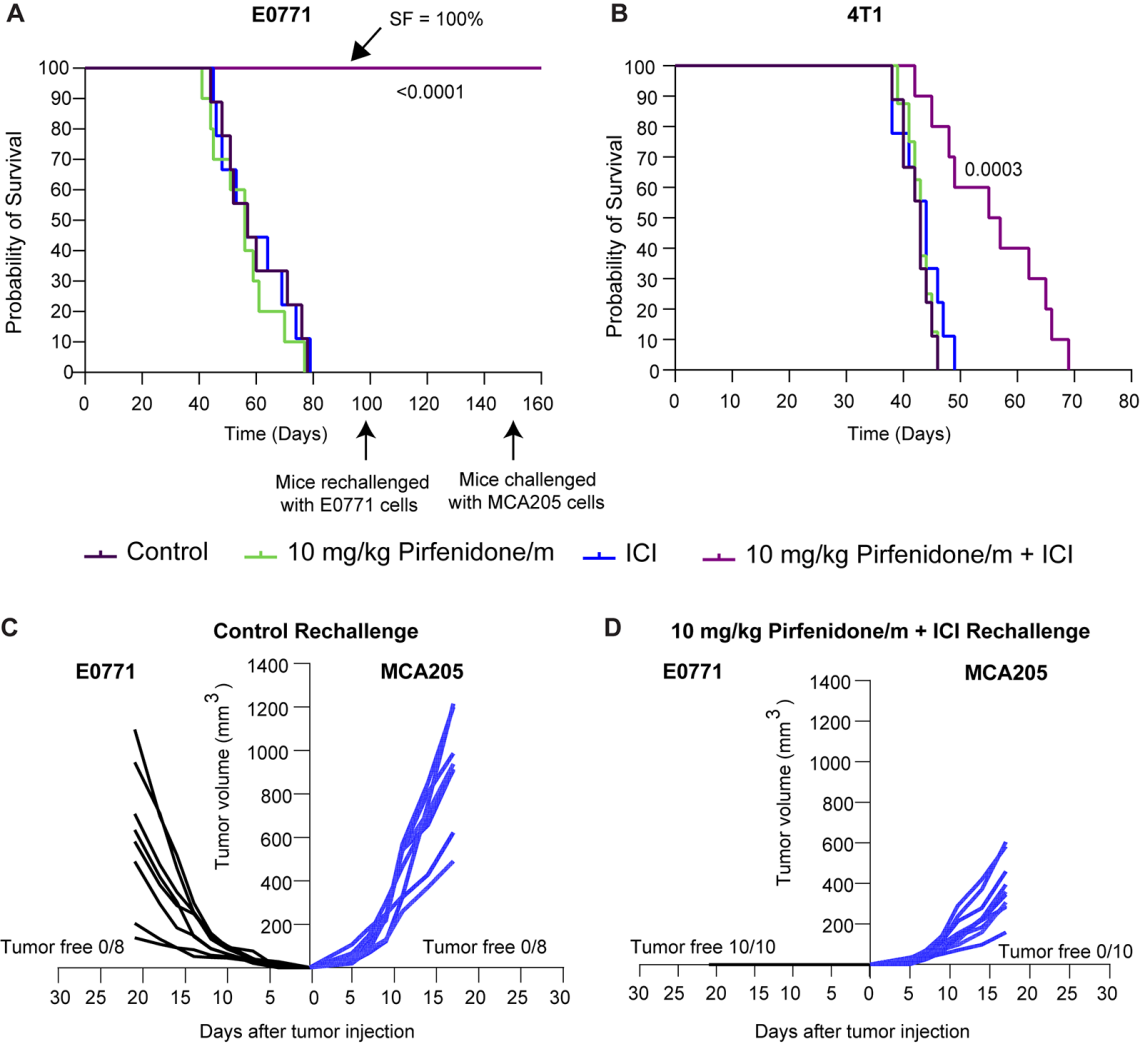
Combination of pirfenidone/m and immunotherapy improves overall survival of mice and induces immunological memory. Kaplan–Meier survival curves for the various treatments considered in (A) E0771 and (B) 4T1 tumors. Statistical analysis was performed by a log-rank test (Mentel–Cox) comparing pirfenidone/m-ICI combination with all other treatment groups. On day 100, survivors of the pirfenidone/m-ICI (*n* = 10) group were rechallenged with 5 × 10^4^ E0771 cells in the site opposite to the first injection. A group of naïve mice of the same age was challenged in parallel to serve as a control. On day 150, mice that remained tumor-free and a group of naïve mice were simultaneously exposed to the irrelevant MCA205 fibrosarcoma cell line (2.5 × 10^5^ cells) through injection in the leg muscle. Individual growth curves of (C) naïve mice (D) pirfenidone/m-ICI group challenged with E0771 (left) and MCA205 (right) tumor cells. The count of mice free from tumors is also depicted in each study.

Our data strongly suggest that the combination of pirfenidone/m with ICIs improves the therapeutic outcomes. To investigate whether this synergy correlates also with improved survival, we surgically removed primary tumors following the completion of the treatment protocol. The goal was to evaluate animal survival in the presence of potential spontaneous metastases that develop during the course of treatment. We found that the combined treatment of pirfenidone/m and ICI cocktail significantly extended the survival of mice in both cancer cell lines, whereas ICI alone had no effect (Figure 7A,B). Particularly, for the E0771 tumors, all mice receiving the combinatorial treatment survived. On day 100, all surviving mice from the pirfenidone/m+ICI along with a group of eight naïve mice were challenged with E0771 cells (Figure 7C,D). As anticipated, all mice in the naïve group did not survive, while the mice in the combination treatment group effectively rejected E0771 cells. Tumor-free mice were challenged again on day 150 with an injection of 2.5 × 10^5^ cells from the unrelated MCA205 fibrosarcoma cancer cell line. In parallel, a group of naïve mice was subjected to the same challenge. Notably, none of the mice in either group managed to reject this irrelevant tumor cell line, highlighting the tumor antigen specificity of the acquired long-term immune memory (Figure 7C,D). Nonetheless, in the pirfenidone/m-ICI group, the tumor growth of fibrosarcoma cancer cells was slower compared to naive mice.

## Conclusions

It has been widely accepted that abnormalities in the TME hinder therapeutic efficacy. In particular, fibrotic tumors, such as breast and pancreatic cancers and sarcomas are resistant to chemo- and immunotherapy because of physical or mechanical abnormalities that interfere with drug delivery and immune cell function.^7,11,33,34^ Determination of the causes of mechanical forces on tumor blood vessels and the consequences of blood vessel collapse on cancer treatment led^4,14^ toward the development of the mechanotherapeutic class of drugs. Importantly, the losartan study led to a phase II clinical trial, which concluded that when losartan is combined with FOLFIRINOX, 60% of unresectable pancreatic tumors become eligible to be resected, thereby making it a “potentially curable” treatment.^2^ These successes demonstrate the clinical potential of mechanotherapeutics to improve cancer therapy, rendering losartan as the “gold standard”.

However, because of the potent antihypertensive properties of losartan, other drugs with mechanotherapeutic properties are being tested in clinical trials, such as the antihistamine ketotifen (EudraCT number: 2022-002311-39). In addition, incorporation of the drug in a nanoparticle formulation can significantly improve its pharmacokinetic properties and, thus, improve safety of administration by reducing its required dose. Here, we developed and thoroughly tested in mouse breast tumor and fibrosarcoma models the efficacy of a nanoformulation of pirfenidone. We found that encapsulation of pirfenidone in a polymeric micelle could be equally effective to the free agent at a 50- to 100-fold reduced dose. Pirfenidone is an approved drug for idiopathic pulmonary fibrosis, and reducing the required pirfenidone dose to modulate the TME by 100-fold can significantly improve the therapeutic window of the drug. We should note that the pH release of pirfenidone is only marginal, which is due to the small number (6) of the pH-responsive DEAEMA moieties that are present within the backbone of the [BzMA_36_-*co*-DEAEMA_6_]-*b*-HEGMA_89_ copolymer chains, which are randomly distributed within the BzMA_36_-*co*-DEAEMA_6_ core-forming copolymer segment. Consequently, by increasing the number of the DEAEMA groups, it is expected that the pH effect will become more pronounced.^35^

Further establishing the use of mechanotherapeutics in cancer therapy requires monitoring their effects during treatment for the development of patient-specific treatment strategies. Toward this direction, we showed the ability of ultrasound imaging, particularly, shear wave elastography for monitoring changes in tissue stiffness and contrast-enhanced ultrasound for monitoring the degree of tumor perfusion, to determine the proper dose of pirfenidone and the duration of treatment prior to administration of ICIs. This work compliments previous research for the use of atomic force microscopy to determine changes in tissue mechanical properties during mechanotherapeutic treatment by taking tumor biopsies at different time points.^36^ Ultrasound imaging, however, has the advantage of being not invasive; it can be more easily processed, and ultrasound elastography is already employed for diagnosis of other diseases, such as liver fibrosis. In addition, testing the effects of the pirfenidone/m in other models of cancer, such as transgenic or PDX models, could further confirm its efficacy to reprogram the TME and assist its clinical translation. In conclusion, our study demonstrates the promise of the use of nanotechnology and ultrasound imaging to consolidate mechanotherapeutics for the patient-specific treatment of fibrotic tumors.

## Methods/Experimental

### Cell Culture

The 4T1 (ATCC CRL-2539) and E0771 (94A001, CH3 BioSystems) breast adenocarcinoma cell lines were maintained in Dulbecco’s modified Eagle medium (DMEM, LM-D1109, Biosera) and Roswell Park Memorial Institute medium (RPMI-1640, LM-R1637, Biosera), respectively, and supplemented with 10% fetal bovine serum (FBS, FB-1001H, Biosera) and 1% antibiotics (A5955, Sigma). Cell lines were preserved in 5% CO_2_ at 37 °C. MCA205 fibrosarcoma cells (SCC173, Millipore) were cultured in RPMI-1640 (LM-R1637, Biosera) containing 2 mM l-glutamine (TMS-002-C, Sigma-Aldrich), 1 mM sodium pyruvate (TMS-005-C, Sigma-Aldrich), 10% FBS (FB-1001H, Biosera), 1× nonessential amino acids (TMS-001-C, Sigma-Aldrich), 1% antibiotics (A5955, Sigma), and 1× β-mercaptoethanol (ES-007-E, Sigma).

### Drugs and Reagents

#### Drugs

Pirfenidone (Esbriet, Roche Pharmaceuticals, Switzerland) was dissolved in deionized water upon heating at 60 °C for 30 min. The immune checkpoint inhibitors mouse monoclonal anti-PD-1 antibody (CD279, clone RMP1-14) and mouse monoclonal anti-CTLA-4 antibody (CD152, clone 9D9) were purchased from BioXCell and diluted in InVivoPure pH 7.0 dilution buffer.

#### Reagents

Aluminum oxide activated (Sigma-Aldrich) and CaH_2_ (Merck, 99.9%) were used as received. Benzene (Fluka, ≥99.5%) and ethyl acetate (Scharlau) were stored over CaH_2_ and distilled before polymerizations. Ethanol (Sharlau, 96%), *n*-hexane (LabScan, 99%), phosphate buffer saline (PBS, Biosera, 10×), and deuterated chloroform CDCl_3_ (Sharlau, 99.8 atom % D) were used as received.

Hexa(ethylene glycol) methyl ether methacrylate (HEGMA, Sigma-Aldrich) was initially diluted in tetrahydrofuran (THF, HPLC grade, Scharlau), and the resulting solution was then passed through a basic alumina column for purification. THF was removed under reduced pressure (Heidolph rotary evaporator), and HEGMA was recovered and used in the polymerization processes, without employing additional purification steps. 2-(Diethylamino)ethyl methacrylate (DEAEMA, 95%, Sigma-Aldrich) was initially passed through basic alumina, stored over CaH_2_, and distilled under vacuum at 50 °C before polymerizations. Benzyl methacrylate (BzMA, 96%, Sigma-Aldrich) was stored over CaH_2_ and bubbled with high-purity N_2_ gas for 30 min immediately prior to the polymerization reaction. 2,2′-Azobis(2-methylpropionitrile) (AIBN, 95%, Sigma-Aldrich), which was employed as the radical initiator, was purified by recrystallization in ethanol. The recrystallization process was performed twice. 2-Cyano-2-propyl benzodithioate (Cyano-CTA, >97%, Sigma-Aldrich) was employed as the chain transfer agent (CTA) without further purification.

Pirfenidone (Esbriet capsules, 267 mg) was recrystallized prior to use as follows: Esbriet powder (0.3192 g) was placed in a 25 mL round-bottom flask followed by the addition of ethanol (16 mL) and stirring for 7 days at room temperature under dark conditions. The resulting solution was filtered to remove the insoluble solids. Afterward, the filtrate was transferred into a 25 mL round-bottom flask, and the solvent was removed using a rotary evaporator. A pale-yellow oil was produced, which was placed in a vacuum oven for 4 h to dry, resulting in a white-yellow powder.

### Polymer Synthesis and Characterization

Polymer synthesis was performed using reversible addition–fragmentation chain transfer (RAFT)-controlled radical polymerization. RAFT is a highly versatile polymerization method enabling the facile synthesis of polymers of various chemical compositions and architectures having well-defined structural characteristics.^37−39^ The synthesis of the targeted [BzMA_*x*_-*co*-DEAEMA_*y*_]-*b*-HEGMA_*z*_ diblock copolymer involved two steps. Initially, a BzMA_*x*_-*co*-DEAEMA_*y*_ random copolymer was prepared bearing hydrophobic/aromatic BzMA moieties combined with pH-responsive DEAEMA units. The aforementioned random copolymer was further used as a macro-CTA for HEGMA to obtain the [BzMA_*x*_-*co*-DEAEMA_*y*_]-*b*-HEGMA_*z*_ functional diblock copolymer.

The chemical structure of the produced polymers prepared in this study was verified by ^1^H NMR spectroscopy using an Avance Bruker 500 MHz spectrometer. ^1^H NMR spectra were recorded in CDCl_3_. Tetramethylsilane (TMS) was used as an internal standard. Their number-average molar mass (*M*_n_^SEC^) was determined by size exclusion chromatography (SEC) using Styragel HR 3 and Styragel HR 4 columns (Polymer Standards Service. The calibration curve used was based on poly(methyl methacrylate) (PMMA) standards.

#### 1 Synthesis of BzMA_*x*_-*co*-DEAEMA_*y*_ Random Copolymer

Cyano-CTA was placed in vacuo for 5 min prior to use. In a 100 mL round-bottom flask maintained under an inert atmosphere (N_2_, 99,999%), Cyano-CTA (147.1 mg, 0.665 mmol) and AIBN (33.8 mg, 0.206 mmol) were dissolved in freshly distilled ethyl acetate (38 mL). The monomers DEAEMA (2.67 mL, 13.3 mmol) and BzMA (9.0 mL, 53.2 mmol) were transferred into the flask, and the resulting solution was degassed by performing three freeze–evacuate–thaw cycles. This was followed by heating at 65 °C for 21 h. The polymerization was stopped by leaving the solution to cool at RT. The random copolymer, the chemical structure of which is provided in Supplementary Figure S14 (9.67 g, 82% polymerization yield, *M*_n_^SEC^: 7538 g mol^–1^, orange color), was precipitated in *n*-hexane and left to dry in vacuo for 24 h. ^1^H NMR (500 MHz, CDCl_3_): δ (ppm) = 0.724–0.914 (b, b′, 3H, br), 1.327–1.952 (a, a′, 2H, br), 3.031–3.058 (e, 4H, br), 3.242–3.302 (d, 2H, br), 4.878 (c′, 2H, br), 5.195 (c, 2H, s), 7.260–7.376 (g–k, 5H, s).

#### 2 Synthesis of [BzMA_*x*_-*co*-DEAEMA_*y*_]-*b*-HEGMA_*z*_ Diblock Copolymer

Chain growth of the initially prepared BzMA_*x*_-*co*-DEAEMA_*y*_ random copolymer was realized by adding HEGMA. The procedure followed for the synthesis of the [BzMA_*x*_-*co*-DEAEMA_*y*_]-*b*-HEGMA_*z*_ diblock copolymer is as follows: The macro-CTA, [BzMA_36_-*co*-DEAEMA_6_] (*M*_n_^SEC^ = 7538 g mol^–1^, 1.50 g, 0.20 mmol), was placed in a 50 mL round-bottom flask, and benzene (14 mL) was added under an inert atmosphere. AIBN (10 mg, 0.062 mmol) dissolved in benzene and HEGMA (5.37 g, 0.0179 mmol) were then transferred into the flask. The reaction mixture was degassed by three freeze–evacuate–thaw cycles and heated at 65 °C for 21 h. The reaction was stopped upon cooling at RT. The obtained solution was condensed using a rotatory evaporator, and the produced diblock copolymer (the chemical structure of which is provided in Supplementary Figure S14, 6.17 g, 87% polymerization yield, *M*_n_^SEC^: 31685 g mol^–1^, pink color) was precipitated in *n*-hexane and dried in a vacuum oven overnight. ^1^H NMR (500 MHz, CDCl_3_): δ (ppm) = 0.720–1.015 (b, b′, b″, 9H, br), 1.720–1.879 (a, a′, a″, 6H, br), 2.153–2.206 (d, e, 6H, s), 3.377 (n, 3H, s), 3.648 (m, l, 4H, s), 4.078 (c′, 2H, br), 4.891 (c, 2H, s), 7.276–7.356 (g–k, 5H, s).

#### 3 Preparation of Diblock Copolymer Micellar Nanocarriers

##### 3.1 Preparation of Pirfenidone-Loaded [BzMA_36_-*co*-DEAEMA_6_]-*b*-HEGMA_89_ Micelles

The diblock copolymer [BzMA_36_-*co*-DEAEMA_6_]-*b*-HEGMA_89_ (MW = 31685 g mol^–1^, 75 mg, 0.0022 mmol) was placed in a glass vial (20 mL) with a screw cap, and it was left to dissolve in PBS (15 mL) under stirring conditions at RT. Subsequently, the solution was filtered twice by using a filter paper. Recrystallized pirfenidone (9 mg, 0.0485 mmol) was mixed with the diblock copolymer micellar solution, and the mixture was placed in the ultrasonic bath for 1 h, followed by stirring at RT in dark conditions. Subsequently, the mixture was filtered using a 0.45 μm cellulose acetate filter, and the filtrate (8 mL) was dialyzed (Slide-A-Lyzer dialysis membrane with a molecular cutoff of 2 kDa) against PBS (1.2 L) to remove any unbound pirfenidone drug. Pirfenidone loading % was determined by means of UV–vis spectrophotometry by recording the UV–vis spectrum of the micellar solution that was recovered after dialysis, at 310 nm (corresponding to pirfenidone). The concentration (and thus the mass) of the encapsulated pirfenidone was determined using the pirfenidone calibration curve (Supplementary Figure S15). This was divided by the initial mass of pirfenidone (9 mg) that was added in the micellar solution (i.e., % loaded pirfenidone = [mass of the encapsulated pirfenidone/initial mass of pirfenidone] × 100), and it was found to be in the range of 90–95%.

##### 3.2 Drug Release Kinetic Studies

The pirfenidone-loaded micellar solution prepared in PBS (pH = 7.2–7.4) that was placed in a dialysis cassette was immersed into (a) PBS solution (pH = 7.2–7.4) and (b) slightly acidic aqueous solution (pH = 6). It was then removed from the cassette at specific time intervals, and after measuring its absorbance (310 nm), it was returned back to the cassette. The process was completed within 3 h.

##### 3.3 Preparation of Pirfenidone/DiR-Loaded [BzMA_36_-*co*-DEAEMA_6_]-*b*-HEGMA_89_ Micelles

Pirfenidone and the fluorescence imaging agent 1,1′-dioctadecyltetramethyl indotricarbocyanine iodide (DiR) were loaded into the [BzMA_36_-*co*-DEAEMA_6_]-*b*-HEGMA_89_ diblock copolymer micelles.^40^ Initially, the block copolymer (75 mg) was dissolved in acetone (12 mL) followed by the addition of pirfenidone (9 mg, 0.0485 mmol). Subsequently, DiR (3 mL from stock solution prepared in acetone, solution concentration: 0.25 g L^–1^) and PBS (15 mL) were added, and the resulting mixture was left to stir overnight in dark conditions at RT, allowing the acetone to evaporate. Insoluble DiR and pirfenidone were removed by centrifugation at 1000*g* for 10 min. The aqueous micellar solution was passed through a cellulose acetate filter (0.45 μm). Unbound DiR and pirfenidone were removed by ultrafiltration in PBS using a Slide-A-Lyzer dialysis tube (molecular cutoff 2 kDa). Both DiR and pirfenidone loading concentrations were determined by recording the UV–vis spectrum of the micellar solution at 750 nm (corresponding to DiR) and 310 nm (characteristic absorption wavelength of pirfenidone), respectively, using the corresponding calibration curves (Supplementary Figure S15).

### Micellar Concentration and Size

The concentration and size distribution of free micellar particles were measured by TRPS using a qNano gold instrument (IZON Science, Oxford, UK). The instrument was set up and calibrated according to the manufacturer’s recommendations. The nanopore membrane NP80 (size range of 40–250 nm) was used and was axially stretched to 47.00 mm. For calibration, CPC100 polystyrene beads (1:1000) (IZON Science, Oxford, UK) were used. The apparatus for both calibration and sample measurements was operated at a voltage of 0.76 V and a pressure equivalent to 20 mbar. Data processing and analysis were performed using the Izon Control Suite software v2.2 (IZON Science Ltd., Oxford, UK).

### In Vitro Cytotoxicity of Pirfenidone-Loaded Micellar Particles

Cell viability was measured using an MTT (3-(4,5-dimethylthiazol-2-yl)-2,5-diphenyltetrazolium bromide) (Sigma-Aldrich, USA) colorimetric assay following the manufacturer’s recommendations. 4T1 cells were plated in a 96-well plate at a density of 1.5 × 10^4^ cells/well. After 24 h, cells were treated in triplicate with different concentrations of free and pirfenidone/m. DMSO was used as a negative control. Treatments were tested at three different time points: 6, 24, and 48 h. At the end of each treatment, 10 μL of MTT was added into each well. After 3 h of incubation at 37 °C the formazan crystals formed were dissolved in 100 μL of DMSO (Sigma-Aldrich, USA), and the absorbance was measured at 570 and 690 nm using a Multiskan FC microplate photometer (Thermo Fisher Scientific, USA). The background absorbance at 690 nm was subtracted. Cell viability was expressed as a percent compared with the untreated control.

### Syngeneic Tumor Models and Treatment Protocols

#### Pirfenidone Dose Response Studies

The syngeneic orthotopic murine breast cancer model was generated by injecting 40 μL of a 5 × 10^4^ 4T1 cell suspension in serum-free medium into the third mammary fat pad of 6–8-week-old female BALB/c mice. Upon reaching an average volume of 150 mm^3^, mice were randomly assigned to five groups (*n* = 8 per group) as follows: control (H_2_O, oral gavage), free pirfenidone 500 mg/kg (oral, gavage), free pirfenidone 5 mg/kg (intravenous injection, i.v.), pirfenidone/m 5 mg/kg (i.v.), and pirfenidone/m 10 mg/kg (i.v.). Mice were treated with pirfenidone daily for 5 days.

#### Antitumor Activity of Immunotherapy in the Orthotopic Breast Tumor Models

Syngeneic, orthotopic tumor models were established through implantation of either 5 × 10^4^ 4T1 or 5 × 10^4^ E0771 murine breast cancer cells in 40 μL of medium without serum into the mammary fat pad of 6-week-old female BALB/c and C57BL/6 mice, respectively. When tumors reached an average size of 150 mm^3^, mice were randomized in the following groups (*n* = 10 per group): H_2_O/IgG (control group, i.p.), pirfenidone/m (10 mg/kg, i.v.), immune checkpoint inhibitors-ICI (cocktail of anti-PD-1, 10 mg/kg, and anti-CTLA-4, 5 mg/kg, i.p.), and pirfenidone/m+ICI. Mice were treated with pirfenidone/m for 3 days (i.e., on days 10, 11, and 12 postimplantation of cancer cells). The ICI cocktail and IgG isotype control were given every 3 days following pretreatment with pirfenidone/m (i.e., days 13, 16 and 19). Mice received a second cycle of pirfenidone/m injections on days 14, 15, 17, and 18. Fibrosarcoma tumors were generated by inoculating female and male C57BL/6 mice at 6 weeks of age (equal number) with 2.5 × 10^5^ MCA205 cells in 50 μL of serum-free medium into the leg muscle. When tumors reached an average size of 150 mm^3^, mice were randomized in the groups described previously (*n* = 10 per group). Mice were treated with pirfenidone/m for 3 days (i.e., on days 8, 9, and 10 postimplantation of cancer cells). The ICI cocktail and IgG isotype control were given every 3 days following pretreatment with pirfenidone/m (i.e., days 11, 14, and 17). Mice received a second cycle of pirfenidone/m injections on days 12, 13, 15, and 16. Two days after completion of the treatment protocol, primary tumors were removed and stored for further analysis.

#### Mice E0771 Rechallenge

Assessment of immunological memory in the pirfenidone/m-ICI study: Tumor-free mice from the pirfenidone/m+ICI (*n* = 10) therapy group were rechallenged after 100 days from the initial tumor injection with E0771 cells in the opposite mammary fat pad (left) and on day 150 with MCA205 fibrosarcoma cells (2.5 × 10^5^) in the leg muscle. Naïve C57BL/6 mice of the same age were also subcutaneously injected with MCA205 or E0771 tumor cells to serve as a control.

The planar dimensions (*x*, *y*) of the tumors were monitored every 2 or 3 days using a digital caliper, and tumor volume was estimated from the volume of a sphere with a diameter equal to the average of planar dimensions. Animal survival was quantified based on the time of death after initiation of treatment.^14^ All in vivo experiments were approved and licensed by the Cyprus Veterinary Services (CY/EXP/PR.L2/2018, CY/EXP/PR.L14/2019, CY/EXP/PR.L15/2019).

### Statistical Analysis

Data are presented as means with standard errors. Groups were compared using one-way or two-way ANOVA with a Dunnett test for multiple comparisons to study statistical significance. Only statistically significant differences along with the exact *P* values are displayed in the figures. A *P* value less than or equal to 0.05 was considered statistically significant.

## Supporting Information Available

Characterization of micelles in vivo, description of ultrasound methods, description of fluorescence immunohistochemistry, supplementary figures (PDF)

## References

[ref1] Sheridan C. Pancreatic cancer provides testbed for first mechanotherapeutics. Nature Biotechnology 2019, 37, 829–831. 10.1038/d41587-019-00019-2. 31375797

[ref2] Murphy J. E.; Wo J. Y.; Ryan D. P.; Clark J. W.; Jiang W.; Yeap B. Y.; Drapek L. C.; Ly L.; Baglini C. V.; Blaszkowsky L. S.; Ferrone C. R.; Parikh A. R.; Weekes C. D.; Nipp R. D.; Kwak E. L.; Allen J. N.; Corcoran R. B.; Ting D. T.; Faris J. E.; Zhu A. X.; et al. Total Neoadjuvant Therapy With FOLFIRINOX in Combination With Losartan Followed by Chemoradiotherapy for Locally Advanced Pancreatic Cancer: A Phase 2 Clinical Trial. JAMA Oncology 2019, 5, 1020–1027. 10.1001/jamaoncol.2019.0892. 31145418 PMC6547247

[ref3] Voutouri C.; Mpekris F.; Papageorgis P.; Odysseos A. D.; Stylianopoulos T. Role of constitutive behavior and tumor-host mechanical interactions in the state of stress and growth of solid tumors. PloS One 2014, 9, e104717 10.1371/journal.pone.0104717. 25111061 PMC4128744

[ref4] Stylianopoulos T.; Martin J. D.; Chauhan V. P.; Jain S. R.; Diop-Frimpong B.; Bardeesy N.; Smith B. L.; Ferrone C. R.; Hornicek F. J.; Boucher Y.; Munn L. L.; Jain R. K. Causes, consequences, and remedies for growth-induced solid stress in murine and human tumors. Proc. Natl. Acad. Sci. U.S.A. 2012, 109, 15101–15108. 10.1073/pnas.1213353109. 22932871 PMC3458380

[ref5] Stylianopoulos T.; Martin J. D.; Snuderl M.; Mpekris F.; Jain S. R.; Jain R. K. Coevolution of solid stress and interstitial fluid pressure in tumors during progression: Implications for vascular collapse. Cancer Research 2013, 73, 3833–3841. 10.1158/0008-5472.CAN-12-4521. 23633490 PMC3702668

[ref6] Padera T. P.; Stoll B. R.; Tooredman J. B.; Capen D.; di Tomaso E.; Jain R. K. Pathology: cancer cells compress intratumour vessels. Nature 2004, 427, 695 10.1038/427695a. 14973470

[ref7] Jain R. K.; Martin J. D.; Stylianopoulos T. The role of mechanical forces in tumor growth and therapy. Annu. Rev. Biomed Eng. 2014, 16, 321–346. 10.1146/annurev-bioeng-071813-105259. 25014786 PMC4109025

[ref8] Jain R. K. Antiangiogenesis strategies revisited: from starving tumors to alleviating hypoxia. Cancer cell 2014, 26, 605–622. 10.1016/j.ccell.2014.10.006. 25517747 PMC4269830

[ref9] Barsoum I. B.; Smallwood C. A.; Siemens D. R.; Graham C. H. A mechanism of hypoxia-mediated escape from adaptive immunity in cancer cells. Cancer Research 2014, 74, 665–674. 10.1158/0008-5472.CAN-13-0992. 24336068

[ref10] Mariathasan S.; Turley S. J.; Nickles D.; Castiglioni A.; Yuen K.; Wang Y.; Kadel E. E.; Koeppen H.; Astarita J. L.; Cubas R.; Jhunjhunwala S.; Banchereau R.; Yang Y.; Guan Y.; Chalouni C.; Ziai J.; Senbabaoglu Y.; Santoro S.; Sheinson D.; Hung J.; et al. TGFbeta attenuates tumour response to PD-L1 blockade by contributing to exclusion of T cells. Nature 2018, 554, 544–548. 10.1038/nature25501. 29443960 PMC6028240

[ref11] Martin J. D.; Seano G.; Jain R. K. Normalizing Function of Tumor Vessels: Progress, Opportunities, and Challenges. Annual Review of Physiology 2019, 81, 505–534. 10.1146/annurev-physiol-020518-114700. PMC6571025 30742782

[ref12] Stylianopoulos T.; Munn L. L.; Jain R. K. Reengineering the Physical Microenvironment of Tumors to Improve Drug Delivery and Efficacy: From Mathematical Modeling to Bench to Bedside. Trends in Cancer 2018, 4, 292–319. 10.1016/j.trecan.2018.02.005. 29606314 PMC5930008

[ref13] Mpekris F.; Panagi M.; Michael C.; Voutouri C.; Tsuchiya M.; Wagatsuma C.; Kinoh H.; Osada A.; Akinaga S.; Yoshida S.; Martin J. D.; Stylianopoulos T. Translational nanomedicine potentiates immunotherapy in sarcoma by normalizing the microenvironment. Journal of Controlled Release 2023, 353, 956–964. 10.1016/j.jconrel.2022.12.016. 36516902

[ref14] Chauhan V. P.; Martin J. D.; Liu H.; Lacorre D. A.; Jain S. R.; Kozin S. V.; Stylianopoulos T.; Mousa A.; Han X.; Adstamongkonkul P.; Popovic Z.; Bawendi M. G.; Boucher Y.; Jain R. K. Angiotensin inhibition enhances drug delivery and potentiates chemotherapy by decompressing tumor blood vessels. Nat. Commun. 2013, 4, 2516 10.1038/ncomms3516. 24084631 PMC3806395

[ref15] Martin J. D.; Panagi M.; Wang C.; Khan T. T.; Martin M. R.; Voutouri C.; Toh K.; Papageorgis P.; Mpekris F.; Polydorou C.; Ishii G.; Takahashi S.; Gotohda N.; Suzuki T.; Wilhelm M. E.; Melo V. A.; Quader S.; Norimatsu J.; Lanning R. M.; Kojima M.; et al. Dexamethasone Increases Cisplatin-Loaded Nanocarrier Delivery and Efficacy in Metastatic Breast Cancer by Normalizing the Tumor Microenvironment. ACS Nano 2019, 13, 6396–6408. 10.1021/acsnano.8b07865. 31187975

[ref16] Mpekris F.; Panagi M.; Voutouri C.; Martin J. D.; Samuel R.; Takahashi S.; Gotohda N.; Suzuki T.; Papageorgis P.; Demetriou P.; Pierides C.; Koumas L.; Costeas P.; Kojima M.; Ishii G.; Constantinidou A.; Kataoka K.; Cabral H.; Stylianopoulos T. Normalizing the Microenvironment Overcomes Vessel Compression and Resistance to Nano-immunotherapy in Breast Cancer Lung Metastasis. Advanced Science 2021, 8, 2001917 10.1002/advs.202001917. 33552852 PMC7856901

[ref17] Panagi M.; Voutouri C.; Mpekris F.; Papageorgis P.; Martin M. R.; Martin J. D.; Demetriou P.; Pierides C.; Polydorou C.; Stylianou A.; Louca M.; Koumas L.; Costeas P.; Kataoka K.; Cabral H.; Stylianopoulos T. TGF-β inhibition combined with cytotoxic nanomedicine normalizes triple negative breast cancer microenvironment towards anti-tumor immunity. Theranostics 2020, 10, 1910–1922. 10.7150/thno.36936. 32042344 PMC6993226

[ref18] Voutouri C.; Panagi M.; Mpekris F.; Stylianou A.; Michael C.; Averkiou M. A.; Martin J. D.; Stylianopoulos T. Endothelin Inhibition Potentiates Cancer Immunotherapy Revealing Mechanical Biomarkers Predictive of Response. Advanced Therapeutics 2021, 4, 2000289 10.1002/adtp.202000289.

[ref19] Polydorou C.; Mpekris F.; Papageorgis P.; Voutouri C.; Stylianopoulos T. Pirfenidone normalizes the tumor microenvironment to improve chemotherapy. Oncotarget 2017, 8, 24506–24517. 10.18632/oncotarget.15534. 28445938 PMC5421866

[ref20] Zhao Y.; Cao J.; Melamed A.; Worley M.; Gockley A.; Jones D.; Nia H. T.; Zhang Y.; Stylianopoulos T.; Kumar A. S.; Mpekris F.; Datta M.; Sun Y.; Wu L.; Gao X.; Yeku O.; Del Carmen M. G.; Spriggs D. R.; Jain R. K.; Xu L. Losartan treatment enhances chemotherapy efficacy and reduces ascites in ovarian cancer models by normalizing the tumor stroma. Proc. Natl. Acad. Sci. U.S.A. 2019, 116, 2210–2219. 10.1073/pnas.1818357116. 30659155 PMC6369817

[ref21] Mpekris F.; Voutouri C.; Baish J. W.; Duda D. G.; Munn L. L.; Stylianopoulos T.; Jain R. K. Combining microenvironment normalization strategies to improve cancer immunotherapy. Proc. Natl. Acad. Sci. U. S. A. 2020, 117, 3728–3737. 10.1073/pnas.1919764117. 32015113 PMC7035612

[ref22] Mpekris F.; Voutouri C.; Panagi M.; Baish J. W.; Jain R. K.; Stylianopoulos T. Normalizing tumor microenvironment with nanomedicine and metronomic therapy to improve immunotherapy. Journal of Controlled Release 2022, 345, 190–199. 10.1016/j.jconrel.2022.03.008. 35271911 PMC9168447

[ref23] Xia T.; He Q.; Shi K.; Wang Y.; Yu Q.; Zhang L.; Zhang Q.; Gao H.; Ma L.; Liu J. Losartan loaded liposomes improve the antitumor efficacy of liposomal paclitaxel modified with pH sensitive peptides by inhibition of collagen in breast cancer. Pharm. Dev Technol. 2018, 23, 13–21. 10.1080/10837450.2016.1265553. 27884084

[ref24] Chauhan V. P.; Chen I. X.; Tong R.; Ng M. R.; Martin J. D.; Naxerova K.; Wu M. W.; Huang P.; Boucher Y.; Kohane D. S.; Langer R.; Jain R. K. Reprogramming the microenvironment with tumor-selective angiotensin blockers enhances cancer immunotherapy. Proc. Natl. Acad. Sci. U.S.A. 2019, 116, 10674–10680. 10.1073/pnas.1819889116. 31040208 PMC6561160

[ref25] Panagi M.; Mpekris F.; Chen P.; Voutouri C.; Nakagawa Y.; Martin J. D.; Hiroi T.; Hashimoto H.; Demetriou P.; Pierides C.; Samuel R.; Stylianou A.; Michael C.; Fukushima S.; Georgiou P.; Papageorgis P.; Papaphilippou P. C.; Koumas L.; Costeas P.; Ishii G.; et al. Polymeric micelles effectively reprogram the tumor microenvironment to potentiate nano-immunotherapy in mouse breast cancer models. Nat. Commun. 2022, 13, 7165 10.1038/s41467-022-34744-1. 36418896 PMC9684407

[ref26] Lutz J. F. Polymerization of oligo(ethylene glycol) (meth)acrylates: Toward new generations of smart biocompatible materials. Polym. Sci, Part A: Polym. Chem. 2008, 46, 3459–3470. 10.1002/pola.22706.

[ref27] Bütün V.; Armes S. P.; Billingham N. C. Synthesis and aqueous solution properties of near-monodisperse tertiary amine methacrylate homopolymers and diblock copolymers. Polymer 2001, 42, 5993–6008. 10.1016/S0032-3861(01)00066-0.

[ref28] Chauhan V. P.; Boucher Y.; Ferrone C. R.; Roberge S.; Martin J. D.; Stylianopoulos T.; Bardeesy N.; DePinho R. A.; Padera T. P.; Munn L. L.; Jain R. K. Compression of pancreatic tumor blood vessels by hyaluronan is caused by solid stress and not interstitial fluid pressure. Cancer Cell 2014, 26, 14–15. 10.1016/j.ccr.2014.06.003. 25026209 PMC4381566

[ref29] Papageorgis P.; Polydorou C.; Mpekris F.; Voutouri C.; Agathokleous E.; Kapnissi-Christodoulou C. P.; Stylianopoulos T. Tranilast-induced stress alleviation in solid tumors improves the efficacy of chemo- and nanotherapeutics in a size-independent manner. Sci. Rep. 2017, 7, 46140 10.1038/srep46140. 28393881 PMC5385877

[ref30] Mpekris F.; Papageorgis P.; Polydorou C.; Voutouri C.; Kalli M.; Pirentis A. P.; Stylianopoulos T. Sonic-hedgehog pathway inhibition normalizes desmoplastic tumor microenvironment to improve chemo- and nanotherapy. Journal of Controlled Release 2017, 261, 105–112. 10.1016/j.jconrel.2017.06.022. 28662901 PMC5548264

[ref31] Voutouri C.; Stylianopoulos T. Accumulation of mechanical forces in tumors is related to hyaluronan content and tissue stiffness. PloS One 2018, 13, e0193801 10.1371/journal.pone.0193801. 29561855 PMC5862434

[ref32] Stylianopoulos T. The Solid Mechanics of Cancer and Strategies for Improved Therapy. Journal of Biomechanical Engineering 2017, 139, 021004 10.1115/1.4034991. PMC5248974 27760260

[ref33] Martin J. D.; Cabral H.; Stylianopoulos T.; Jain R. K. Improving cancer immunotherapy using nanomedicines: progress, opportunities and challenges. Nature Reviews Clinical Oncology 2020, 17, 251–266. 10.1038/s41571-019-0308-z. PMC8272676 32034288

[ref34] Nevala-Plagemann C.; Hidalgo M.; Garrido-Laguna I. From state-of-the-art treatments to novel therapies for advanced-stage pancreatic cancer. Nature Reviews Clinical Oncology 2020, 17, 108–123. 10.1038/s41571-019-0281-6. 31705130

[ref35] Shang Y.; Zheng N.; Wang Z. Tetraphenylsilane-Cored Star-Shaped Polymer Micelles with pH/Redox Dual Response and Active Targeting Function for Drug-Controlled Release. Biomacromolecules 2019, 20, 4602–4610. 10.1021/acs.biomac.9b01472. 31674776

[ref36] Stylianou A.; Mpekris F.; Voutouri C.; Papoui A.; Constantinidou A.; Kitiris E.; Kailides M.; Stylianopoulos T. Nanomechanical properties of solid tumors as treatment monitoring biomarkers. Acta Biomaterialia 2022, 154, 324–334. 10.1016/j.actbio.2022.10.021. 36244596

[ref37] Moad G.; Rizzardo E. A 20th anniversary perspective on the life of RAFT (RAFT coming of age). Polym. Int. 2020, 69, 658–661. 10.1002/pi.5944.

[ref38] Boyer C.; Bulmus V.; Davis T. P.; Ladmiral V.; Liu J.; Perrier S. Bioapplications of RAFT polymerization. Chem. Rev. 2009, 109, 5402–5436. 10.1021/cr9001403. 19764725

[ref39] Krasia T. C.; Patrickios C. Amphiphilic Polymethacrylate Model Co-Networks: Synthesis by RAFT Radical Polymerization and Characterization of the Swelling Behavior. Macromolecules 2006, 39, 2467–2473. 10.1021/ma051747i.

[ref40] Cho H.; Indig G. L.; Weichert J.; Shin H. C.; Kwon G. S. In vivo cancer imaging by poly(ethylene glycol)-b-poly(varepsilon-caprolactone) micelles containing a near-infrared probe. Nanomedicine: Nanotechnology, Biology, and Medicine 2012, 8, 228–236. 10.1016/j.nano.2011.06.009. 21704593 PMC3193583

