# Supplementary material for: Pirfenidone-Loaded Polymeric Micelles as an Effective Mechanotherapeutic to Potentiate Immunotherapy in Mouse Tumor Models
Source: ACS Nano. 2023 Dec 6;17(24):24654–67. doi: 10.1021/acsnano.3c03305 (PMC10753878; doi:10.1021/acsnano.3c03305)
Supplement: Supplementary file 1 — nn3c03305_si_001.pdf [file nn3c03305_si_001.pdf]

Supporting Information

Pirfenidone-loaded polymeric micelles as an effective mechanotherapeutic to potentiate immunotherapy in mouse tumor models

Fotios Mpekris[1+], Petri Ch. Papaphilippou[2+], Myrofora Panagi[1], Chrysovalantis Voutouri[1], Christina Michael[1], Antonia Charalambous[1], Mariyan Marinov Dinev[2], Anna Katsioloudi[3], Marianna Prokopi-Demetriades[3,4], Andreas Anayiotos[4], Horacio Cabral[5], Theodora Krasia-Christoforou[2], Triantafyllos Stylianopoulos[1*]

[1]Cancer Biophysics Laboratory, Department of Mechanical and Manufacturing Engineering, University of Cyprus, 1678 Nicosia, Cyprus

[2] Polymers and Polymer Processing Laboratories, Department of Mechanical and Manufacturing Engineering, University of Cyprus, 1678 Nicosia, Cyprus

[3]Theramir Ltd, 3036 Limassol, Cyprus

[4]Biomechanics and Living Systems Analysis laboratory, Cyprus University of Technology, 3036 Limassol, Cyprus

[5]Department of Bioengineering, Graduate School of Engineering, The University of Tokyo, Bunkyo, 113-8656 Tokyo, Japan

[+]equal contribution

*correspondence to Triantafyllos Stylianopoulos: tstylian@ucy.ac.cy

Blood half-life measurement of pirfenidone micelles. Pirfenidone/m were injected to 4 BALB/c mice (8 weeks, female) through tail vein (10 mg/kg on a pirfenidone basis). At predetermined time points (0.5, 1, 2, 4, 9 and 24 h post-injection), the mice were anesthetized in 3% isoflurane oxygen flow and 50 µl blood sample was collected retro-orbitally and scanned at 20% excitation power for 60 sec at 745 nm excitation and 870 nm emission wavelength using the AMI-HT imaging system.

Accumulation of pirfenidone micelles in orthotopic breast cancer primary tumors. BALB/c female mice bearing orthotopic 4T1 tumors of 100 mm^3 were randomized into two groups (n=4 mice per group) and treated daily with H_2O (control) or 10 mg/kg of Pirfenidone/m for 3 days. On day 6 all mice received an i.v. injection of DiR-labelled Pirfenidone/m (10 mg/kg), anesthetized with isoflurane and scanned at 20% excitation power for 5 sec at 745 nm excitation and 870 nm emission wavelength. Biodistribution of nanoparticles was assessed at 3, 6 and 24 h post-injection using the AMI-HT imaging system.

Conjugation of aPD-1 and aCTLA-4 antibodies to Atto 680-NHS. Atto 680 conjugated antibodies were prepared by conjugating the Atto680 to PD-1 (CD279, clone RMP1-14) and CTLA-4 (CD152, clone 9D9) monoclonal antibodies. Atto 680 NHS ester was dissolved in DMSO 2 mg/ml prior to conjugation. A dye to protein ratio of 7 was used to achieve optimum conjugation. The mixture was incubated at RT for 2 h under constant stirring. Labeled mAb was separated from unreacted dye by gel permeation chromatography using a PD-10 desalting column contain Sephadex G-25 resin according to manufacturer's instructions. The degree of labeling was determined by absorption spectrophotometry.

Accumulation of immunotherapy in orthotopic breast cancer primary tumors. BALB/c female mice bearing orthotopic 4T1 tumors of 100 mm^3 were randomized into two groups (n=4 mice per group) and treated daily with H_2O (control) or 10 mg/kg of Pirfenidone/m for 3 days. On day 4 all mice received an i.v. injection of conjugated antibodies and 6h later mice were sacrificed and tumors were removed and scanned at 20% excitation power for 5 sec at 675 nm excitation and 790 nm emission wavelength using the AMI-HT imaging system.

Interstitial fluid pressure. Interstitial fluid pressure (IFP) was measured *in vivo* using the previously described wick-in-needle technique after mice were anesthetized with i.p. injection of Avertin and prior to tumor excision[1].

Tumor stiffness and perfusion monitoring: The elastic modulus and perfusion of tumors were monitored with ultrasound shear wave elastography (SWE) and contrast-enhanced ultrasound (CEUS), respectively.

SWE was employed on a Philips EPIQ Elite Ultrasound system using a linear array transducer (eL18-4), according to previous research[2]. The method generates a shear wave velocity via an acoustic push pulse, creating a color mapped elastogram (in kPa) where red indicates hard and blue soft tissue. A confidence display is also used as a reference of the shear wave quality of the user-defined region of interest (ROI). The average value of the tumor region is automatically generated by the system under default scanner settings and expressed in kPa. The settings that were used were: frequency 10 MHz, power 52%, B-mode gain 22 dB, dynamic range 62 dB. SWE was performed at two different planes of each tumor and the average value of both planes was used for our analysis.

CEUS was employed to assess tumor associated vascular perfusion after bolus injection of contrast agents (SonoVue 8 μl of sulphur hexafluoride microbubbles encapsulated by a phospholipid shell with a mean diameter of 2.5 μm, retro-orbital administration). Ultrasound scanning of tumors was performed using the linear array transducer L12-5. Contrast first harmonic signals were received at 8 MHz with a mechanical index of 0.06. For all subjects, the depth of the focus was set to 3 cm allowing measurements of the full depth of the tumor. Gain was set at 90% for each recording. Focus was optimized and standardized for each subject when finding the tumor area using B-mode imaging. Real-time power modulation imaging was initiated after flashing imaging with a high mechanical index to destroy the microbubbles in tumor tissue to peak contrast intensity to allow visualization of bubble replenishment. Image analysis was performed offline using an ultrasound quantification and analysis software (QLAB, Phillips Medical Systems). From the produced time intensity curves, we used as measures of perfusion the time required to reach the peak intensity-rise time (RT), which is related to blood flow[3, 4, 5]. Prior to each ultrasound application, mice were

anesthetized by i.p. injection of Avertin (200 mg/kg) and ultrasound gel was applied to the imaging region to prevent any pressure of the transducer on the underlying tissue.

Fluorescence immunohistochemistry. Tumors were excised, washed in 1x PBS twice for 10 min and incubated in 4 % PFA at 4 °C for 24 h. The samples were washed twice in 1x PBS for 10 min, dehydrated in successive steps of alcohol and xylene, and finally embedded in paraffin. Serial sections (7 μm) of paraffin-embedded tissues were produced using the microtome (Accu-Cut SRM 200 Rotary Microtome, SAKURA). The sections were flattened out into water and allowed to dry overnight at 37 °C, deparaffinized, and rehydrated.

Hyaluronan binding protein staining and quantification. Antigen retrieval was performed on tissue sections (microwave heat treatment with TriSodium Citrate, pH 6, for 20 min), washed with 1x TBS/ 0.025 % Triton X-100 (TBS-T), and incubated in blocking solution (2 % BSA, 0.2 % Triton 100x) for 2 h at room temperature (RT). Afterwards, slides were incubated with biotinylated hyaluronan binding protein (b-HABP, AMS.HKD-BC41, amsbio 1:100) overnight at 4 °C. Next, the sections were incubated with streptavidin-FITC conjugate (SA1001, Invitrogen 1:1000) and DAPI nuclear stain at RT for 2 h in the dark. For all stained tissue sections, confocal z-stacks were acquired with 1 μm z-spacing on a Leica STELLARIS microscope using a 20x objective. The entire tissue section was imaged using the spiral-scan function of LAS X Navigator. Quantification of the area fraction of hyaluronan was quantified as the number of pixels of immunofluorescence image with signal intensity above a threshold determined using a control slide without primary antibody normalized to the number of pixels positive for DAPI stain.

Collagen staining and quantification. Tissue sections were deparaffinized and rehydrated. They were then submerged in Picrosirius red for 1 h at RT. Next, tissue sections were rinsed in two changes of acetic acid followed by absolute ethanol. Then, they were mounted with DPX mountant for histology (Sigma-Aldrich). Area fraction was determined as the red area, which represents collagen fibers, divided by the total tissue area. Images from the tumor interior and periphery were acquired at 20x magnification using an Olympus BX53 microscope. To enable quantification,

images of the same staining were taken at identical settings. The images were analyzed using custom and built-in algorithms in MATLAB (MathWorks, Inc., Natick, MA, USA).

CAFs staining and quantification. CAFs were identified with αSMA. The proliferation marker Ki67 was also investigated to determine the fraction of proliferating CAFs. Antigen retrieval and incubation with blocking solution (10 % FBS, 3 % donkey serum, 0.05 % Tween-20 in 1x PBS) for 1 h at RT was performed on tissue sections. Following washing with TBS-T (0.25 % Tween-20), tissue sections were incubated in blocking solution for endogenous mouse IgG (ab6668, Abcam 1:100) for 1 h at RT. Then tissue sections were stained for αSMA (ab7817, Abcam 1:50) and Ki67 (ab15580, Abcam 1:200) overnight at 4 °C and signal was detected by Alexa Fluor-647 anti-rabbit IgG (H+L) (A21244, Invitrogen 1:400) and -488 anti-mouse IgG (H+L) (A21202, Invitrogen 1:400) secondary antibodies and cell nuclei were stained with DAPI (Sigma-Aldrich, 1:100 of 1 mg/mL stock) for 2 h at RT. They were then mounted on microscope slides using the ProLong gold antifade mountant (Invitrogen) and covered with a glass coverslip. Quantification of the area fractions of CAFs and Ki67 were quantified separately as the number of pixels of immunofluorescence image with signal intensity above a threshold determined using a control slide without primary antibody normalized to the number of pixels positive for DAPI stain. Then, the fraction of proliferating CAFs were determined using the number of pixels with colocalized signal (αSMA and Ki67) divided by the total number of αSMA^+ pixels.

Pericyte coverage staining and quantification. Pericytes were identified either with αSMA/CD31 colocalization. Briefly, tissue cryosections were cut to 10 μm and blocked (10 % FBS, 3 % donkey serum, 0.3 % Tween-20 in 1x PBS) overnight at 4 °C. αSMA was double-stained with CD31. The tissue sections were incubated with rabbit anti-αSMA (ab5694, Abcam 1:100) and rat anti-CD31 (553370, BD Pharmingen, 1:50) overnight at 4 °C. After washing, tissue sections were then incubated with Alexa Fluor-488 anti-rabbit IgG (H+L) (A11034, Invitrogen 1:400) and -647 anti-rat IgG (H+L) (A21247, Invitrogen 1:600) secondary antibodies and DAPI for 1 h at RT. The areas positive for pericyte marker (αSMA) and endothelial cell marker (CD31) were quantified separately as pixels of the immunofluorescence image with signal intensity above

a threshold determined using a control slide without primary antibody. Then, pericyte coverage fraction was determined using the number of pixels with colocalized signal (αSMA$^+$ and CD31$^+$) divided by the total number of CD31$^+$ pixels.

CD8 staining and quantification. T cells were identified with CD8. The proliferation marker Ki67 was also investigated to determine the fraction of proliferating T cells. 7 μm thick tissue sections were deparaffinized and rehydrated. Antigen retrieval was performed, with tissue sections microwaved in Tris-EDTA pH 9 for 20 min. Tissue sections were then incubated with blocking solution (TBS-T, 10% FBS, 3% goat serum) overnight at 4 °C. They were then incubated with Ki67 antibodies (ab15580, Abcam 1:200) and CD8 antibodies (14-0808-82, eBioscience 1:100) for 2 hr at RT. The tissue sections were then washed twice with TBS-T (0.025% Tween-20). They were then incubated with Alexa Fluor-647 anti-rat IgG (H+L) (A21247, Invitrogen 1:400) and -488 anti-rabbit IgG (H+L) (A11034, Invitrogen 1:400) secondary antibodies and DAPI for 1 hr at RT. The areas positive for CD8 and Ki67 were quantified separately as pixels of the immunofluorescence image with signal intensity above a threshold determined using a control slide without primary antibody. Then, fraction of proliferating CD8$^+$ T cells was determined using the number of pixels with colocalized signal (CD8$^+$ and Ki67$^+$) divided by the total number of CD8$^+$ pixels.

T cell proximity to tumor vessels. To assess the proximity of T cells to tumor vessel capillaries, we performed immunofluorescence staining for the CD31 endothelial marker and surface CD3 protein, a pan-T cell marker. Briefly, paraffin-embedded 4T1 tumor tissue sections were deparaffinized, rehydrated and subjected to antigen retrieval with sodium citrate buffer pH 6. Slides were washed with TBS-T and incubated in blocking solution (15 % FBS, 1.5% BSA and 0.3 % Tween-20 in TBS) overnight at 4 °C. Then, tissue sections were incubated with rabbit anti-CD31 (EPR17259, Abcam 1:100) and rat anti-CD3 (ab11089, Abcam 1:100) primary antibodies, overnight at 4°C. CD31 signal was detected with Alexa Fluor-488 anti-rabbit IgG (H+L) (A11034, Invitrogen 1:400) and CD3 signal with CF555 anti-Rat IgG (H+L) (20233, biotium, 1:400) secondary antibodies for 2 h in the dark. Cell nuclei were stained with DAPI. Association of T

cells on blood vessels was quantified by the adjacent CD3-CD31 pixels over the total CD3 pixels to the number of DAPI positive pixels.

PD-L1 staining and quantification. Paraffin tissue sections 7 μm thick were deparaffinized, rehydrated and subjected to antigen retrieval with sodium citrate buffer pH 6. Slides were washed with TBS-T and incubated in blocking solution (15 % FBS, 1.5% BSA and 0.3 % Tween-20 in TBS) overnight at 4 °C. Then, tissue sections were incubated with the primary antibody for PD-L1 (clone D5V3B, 64988, Cell Signaling, 1:100) and allowed to bind overnight at 4 °C. Tissue sections were then washed and incubated with Alexa Fluor-488 anti-rabbit IgG (H+L) (A11034, Invitrogen 1:400) and DAPI for 2 h at RT. Quantification of the area fraction of PD-L1+ cells was determined as the number of pixels of immunofluorescence image with signal intensity above a threshold determined using a control slide without primary antibody normalized to the number of pixels positive for DAPI stain.

Flow cytometry. On day 21 of treatment, E0771 breast tumors (n=6-10 per treatment group) were harvested in cold RPMI, minced into fine fragments and incubated with 1 ml reconstituted Liberase (0.5 mg per tumor, Roche) for 30 min at 37 ^{o}C on an end-over-end shaker. Enzymatic digestion was ceased by the addition of RPMI media containing 10% FBS and 1% antibiotic/antimycotic solution. The resulting tissue homogenates were filtered through 40 μm cell strainers and single cell suspensions were collected and counted. Cell suspensions were then incubated with fixable viability dye (Invitrogen) for gating of viable cells. Non-specific antibody binding was blocked following incubation with the rat anti-mouse CD16/CD32 mAb (BD Bioscience) for 10min at RT. $1x10^6$ cells per sample were labeled with the various fluorochrome conjugated antibodies, washed and resuspended in 0.5% BSA, 1x PBS buffer. The anti-mouse antibodies used in the experiment are the following; CD4-PerCP-Cy5.5 (GK1.5, BioLegend), CD8a-V450 (53-6.7, eBioscience), Foxp3-FITC (FJK-16s, Invitrogen), CD45-V500 (30-F11, BD Bioscience), CD25-PE-Cy7 (PC61.5, BD Bioscience), CD3-PE (145-2C11, BD Bioscience), CD11b-e450 (M1/70, eBioscience), F4/80-APC (BM8, BioLegend), CD206-PE-Cy7 (C068C2, BioLegend), CD127-APC (A7R34, BioLegend), Gr-1-PE (RB6-8C5, BioLegend), CD38-PerCP-Cy5.5 (90,

BioLegend). Flow cytometry data were obtained using BD FACSLyric III flow cytometer and analyzed using BD FACS Suite software. Data presented is representative of singlets, live cells.

Supplementary Figures

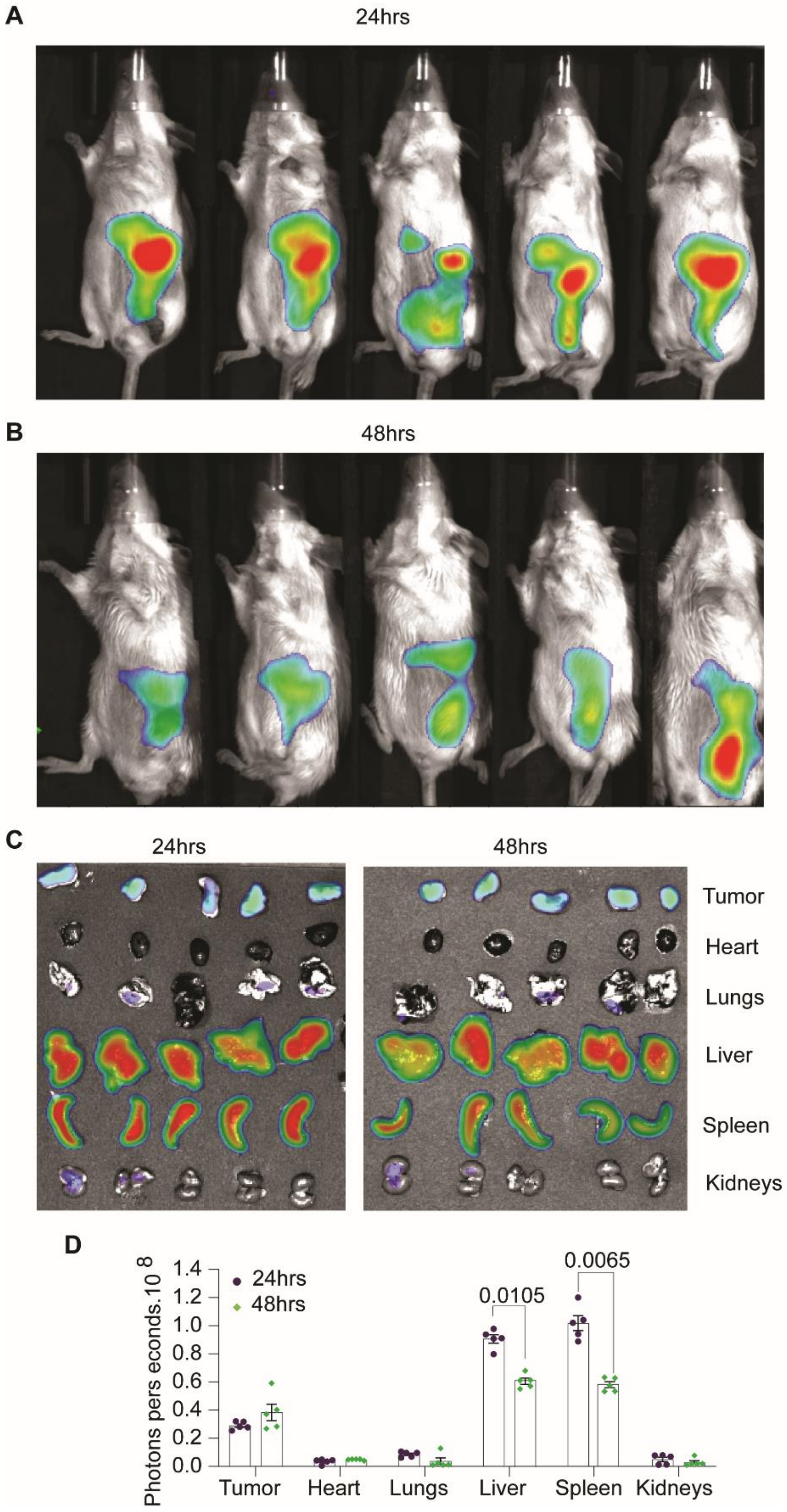

Supplementary Figure S1. Micelles distribution after (A) 24hrs and (B) 48hrs in mice after one injection of Pirfenidone/DiR-micelles. (C) Ex-vivo imaging of micelles distribution in different organs after 24hrs and 48hrs after one injection of Pirfenidone/DiR-micelles. (D) Quantification of signal from ex-vivo imaging of different organs and tumors at 24hrs and 48hrs. Data are presented as mean ± SE. Statistical analyses were performed by using two-way ANOVA with multiple comparisons Dunnett test.

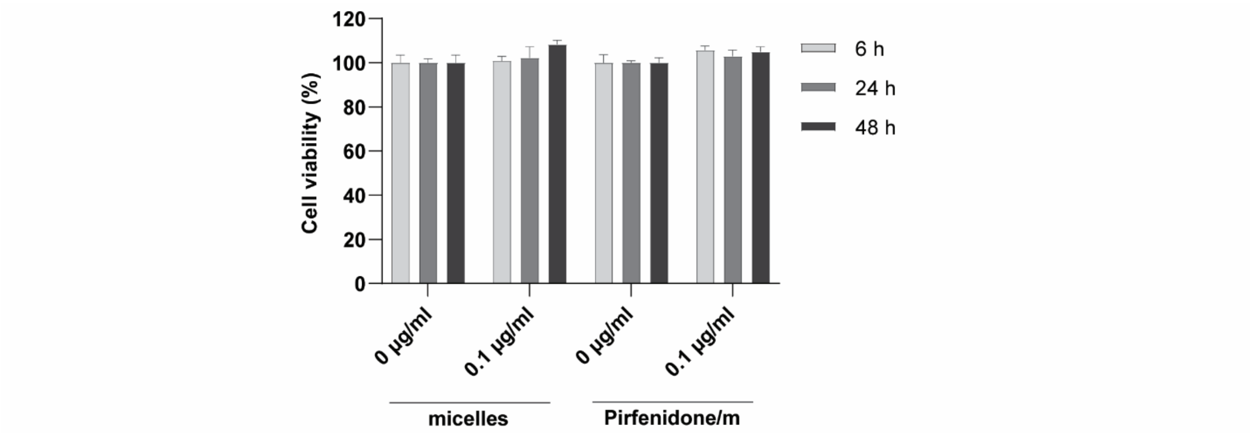

Supplementary Figure S2. Mitochondrial activity assessed by MTT assay of 4T1 cells treated with the polymeric micelles and the pirfenidone loaded-polymeric micelles for 6, 24 and 48 h. concentration of pirfenidone in micelles is 0.7g/L All data presented as mean ± SE, n = 3.

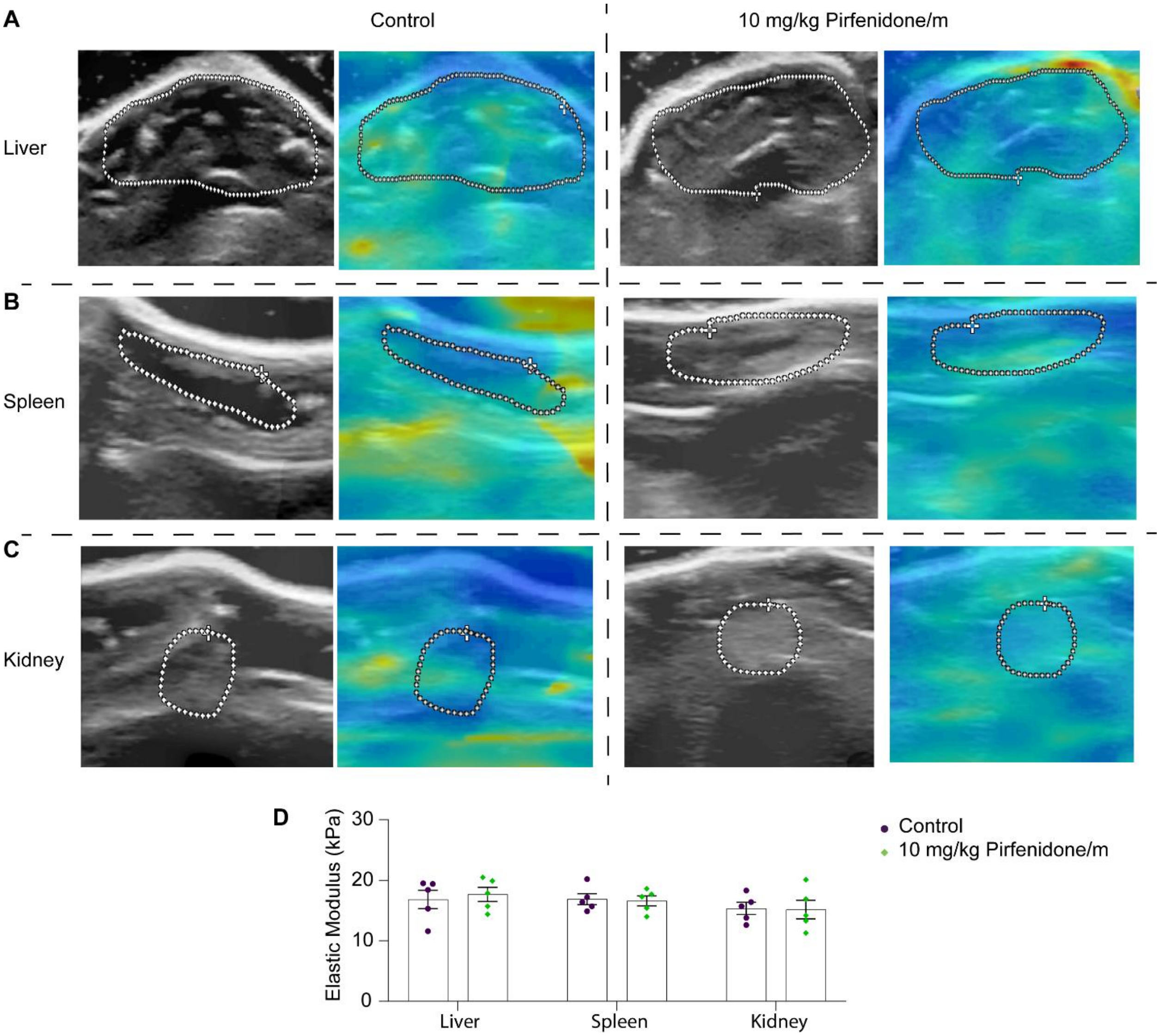

Supplementary Figure S3. **Pirfenidone micelles do not affect stiffness of healthy organs.** Representative B-mode and SWE images for (A) Liver, (B) Spleen and (C) Kidney tissues. (D) Elastic modulus values measured with SWE (n = 3 mice, N=2 image fields per mouse)

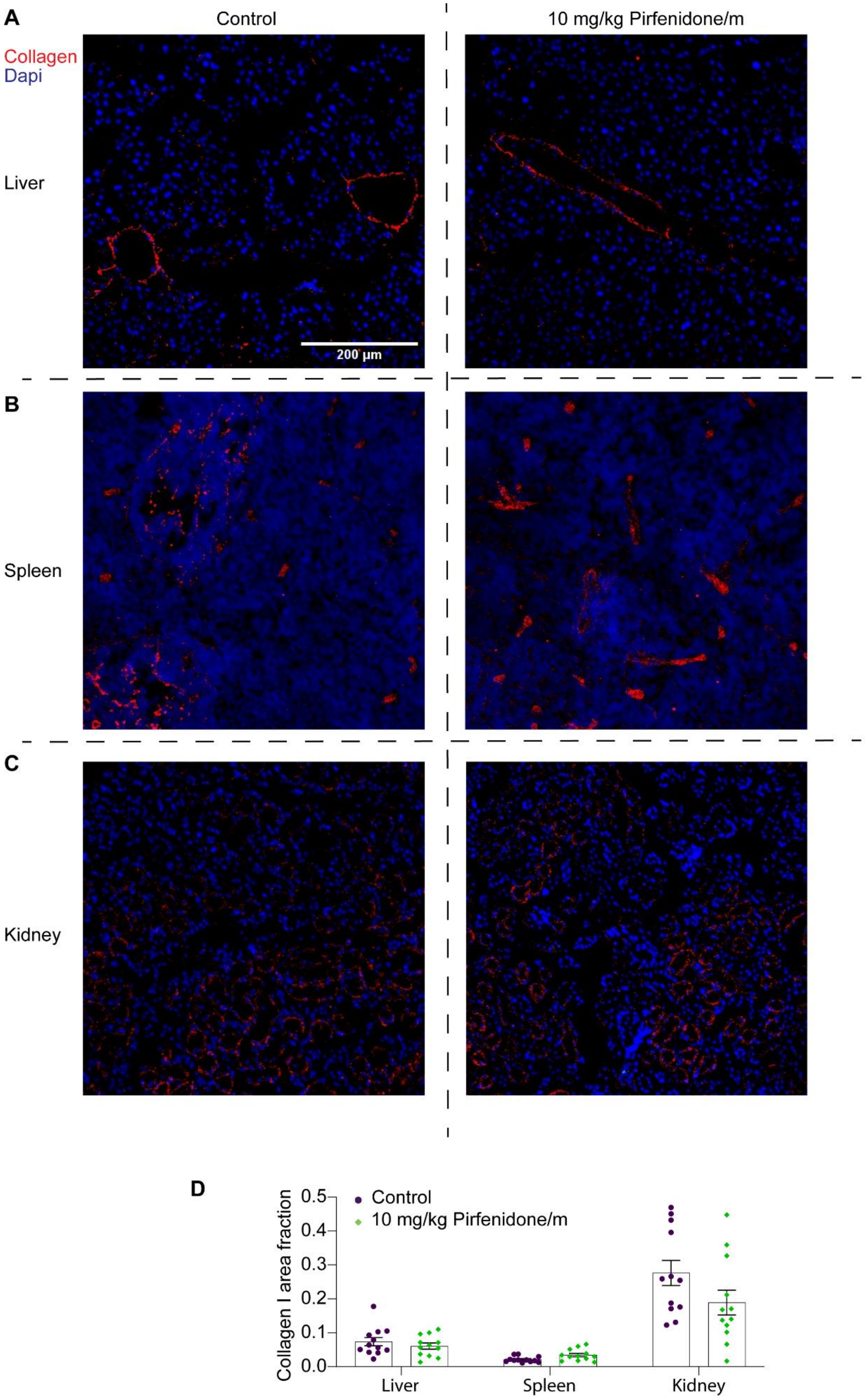
A
Control
10 mg/kg Pirfenidone/m
Collagen
Dapi
Liver
200 µm
B
Spleen
C
Kidney
D
Collagen I area fraction
Control
10 mg/kg Pirfenidone/m
0.0
0.1
0.2
0.3
0.4
0.5
Liver
Spleen
Kidney

***Supplementary Figure S4.* Pirfenidone micelles dot not affect collagen levels of healthy organs.** Representative light microscope images of Collagen I staining (red color) counterstained with nuclear staining (blue) for (A) Liver, (B) Spleen and (C) Kidney tissue. White scale bar indicates 0.2 mm. (D) Graph of the area fraction of Collagen I indicating collagen fibers in tissue sections (n = 3 mice, N = 4 image fields).

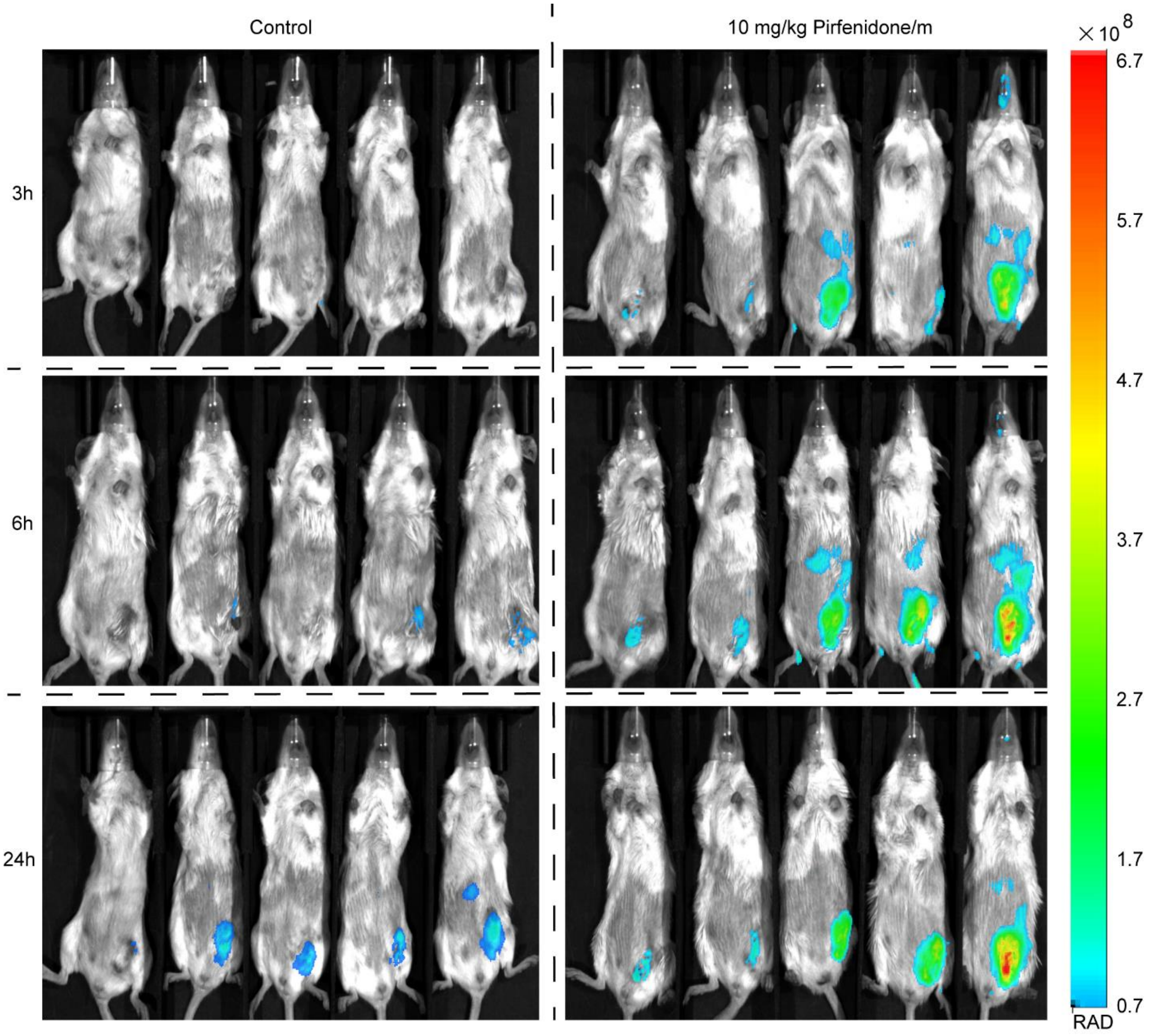

***Supplementary Figure S5*. Pirfenidone micelles increase drug delivery in 4T1 tumors.** Distribution of DiR-labelled micelles in control and Pirfenidone/m treated mice.

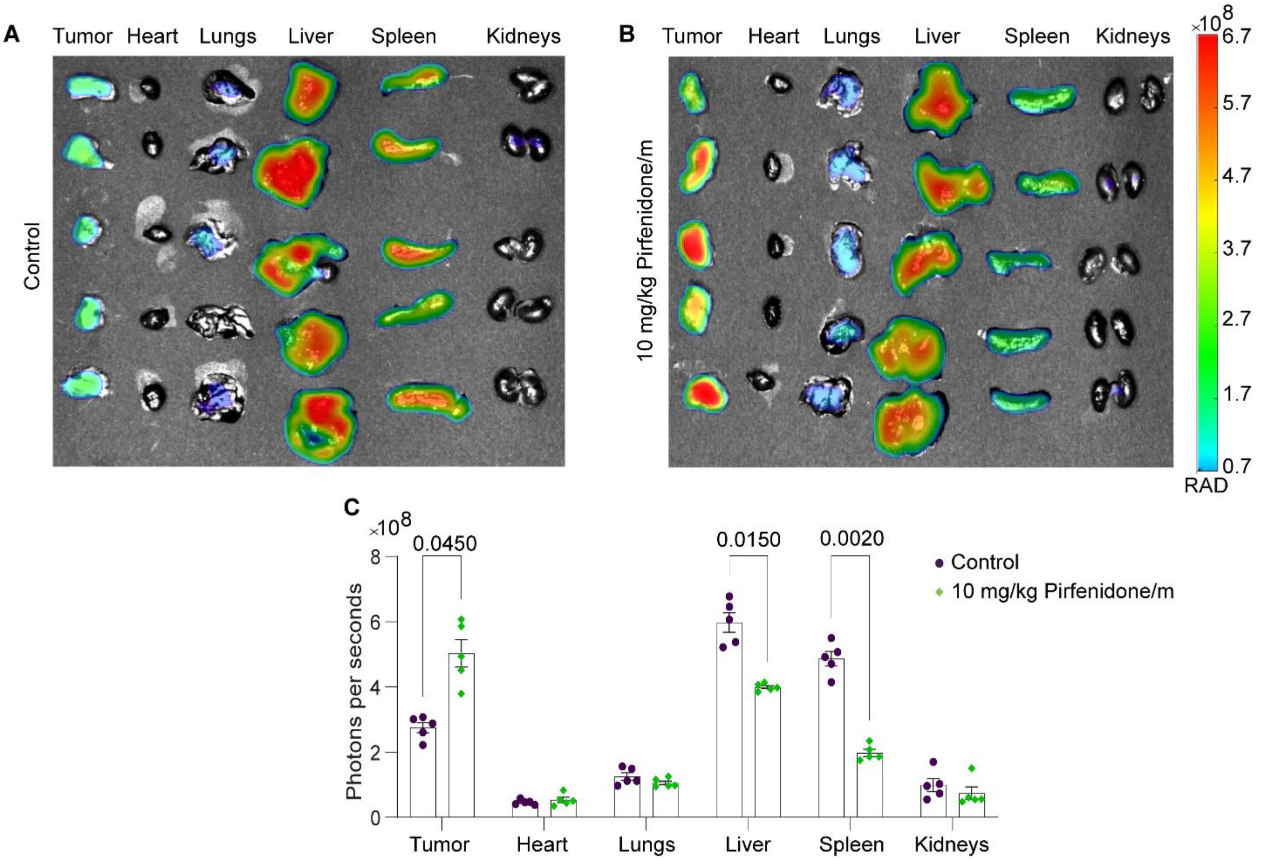

Supplementary Figure S6. **Pirfenidone micelles improve drug delivery in 4T1 tumors but not in other organs.** Ex-vivo imaging of micelles distribution in different organs in (A) control and (B) Pirfenidone/m treated mice after injection with DiR-micelles at 24h. (C) Quantification of signal from ex-vivo imaging of different organs and tumors at 24h. Data are presented as mean ± SE. Statistical analyses were performed by using two-way ANOVA with multiple comparisons Dunnett test.

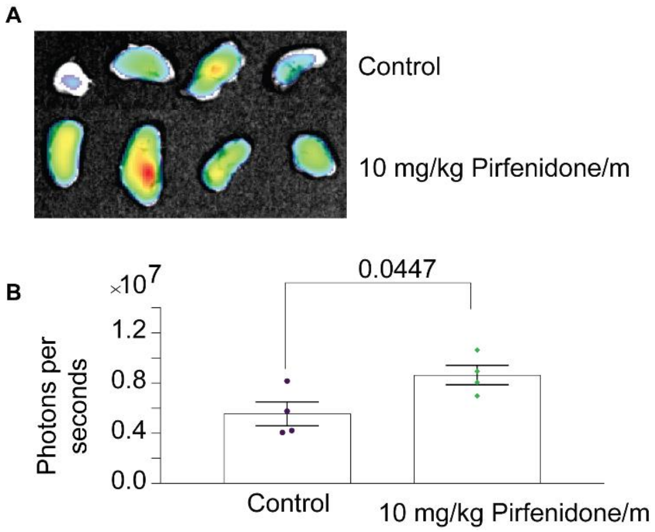

Supplementary Figure S7. **Pirfenidone micelles significantly increase delivery of fluorescent immunotherapy in 4T1 tumors.** (A) Ex-vivo imaging of immunotherapy distribution in tumors in control and Pirfenidone/m treated mice after injection with fluorescent ICI at 6h. (B) Quantification of signal from ex-vivo imaging of tumors at 6h. Data are presented as mean ± SE. Statistical analyses were performed by using for ordinary one-way ANOVA with multiple comparisons Dunnett test.

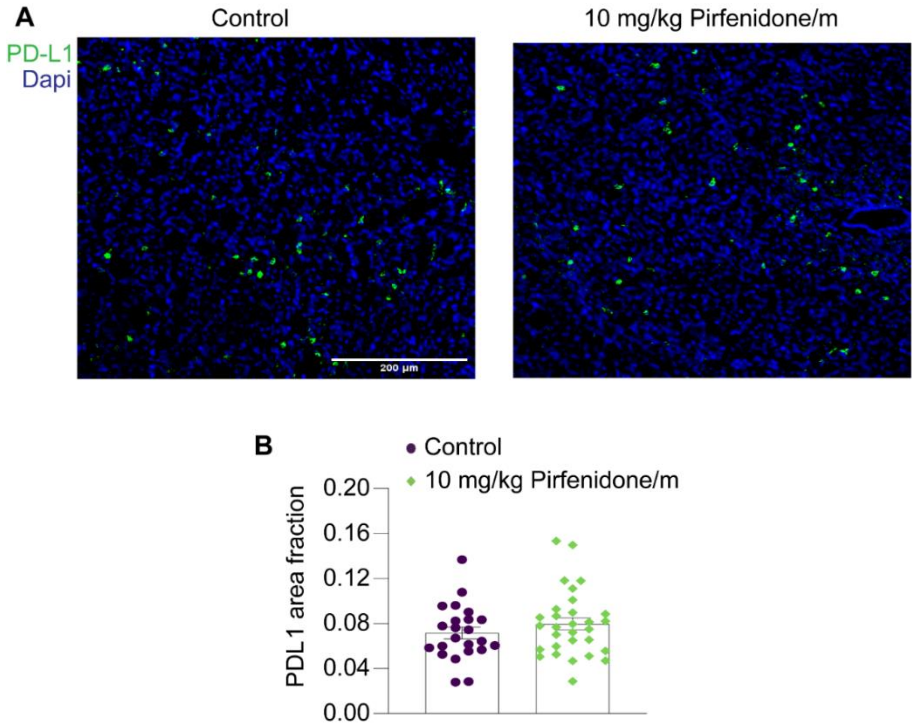

Supplementary Figure S8. **Efficacy of Pirfenidone micelles in PD-L1 protein levels**. (A) Representative immunofluorescence images of PD-L1 staining (green) counterstained with nuclear staining (blue). White scale bar indicates 0.2 mm. (B) Graph of the area fraction of PD-L1 staining in immunofluorescence images of 4T1 tissue.

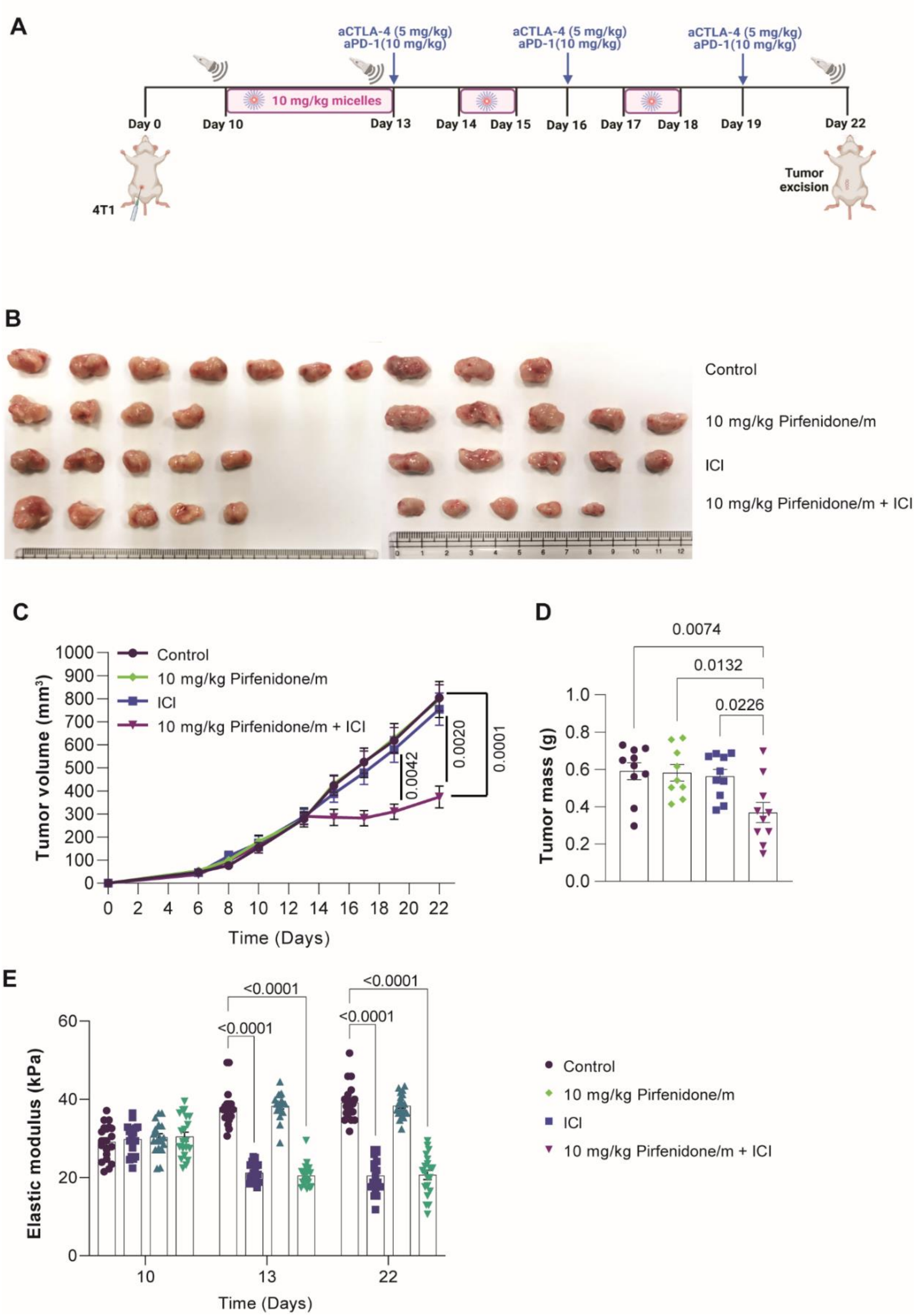

Supplementary Figure S9. **Pirfenidone micelles significantly improve the efficacy of immunotherapy in 4T1 tumors.** (A) Experimental treatment protocol. Created

with BioRender.com. (B) Images of tumors after removal. (C) Tumor growth (n=10 mice), (D) tumor mass (n=10 mice) and (E) tumor elastic modulus (n=10 mice, N=2 image fields per mouse) measured with SWE of 4T1 tumors treated with Pirfenidone/m and anti-CTLA4/anti-PD-1 antibodies. Data are presented as mean ± SE. Statistical analyses were performed by using for (C) mixed-effects analysis with multiple comparisons Tukey test, for (D) ordinary one-way ANOVA with multiple comparisons Dunnett test and for (E) using two-way ANOVA with multiple comparisons Dunnett test.

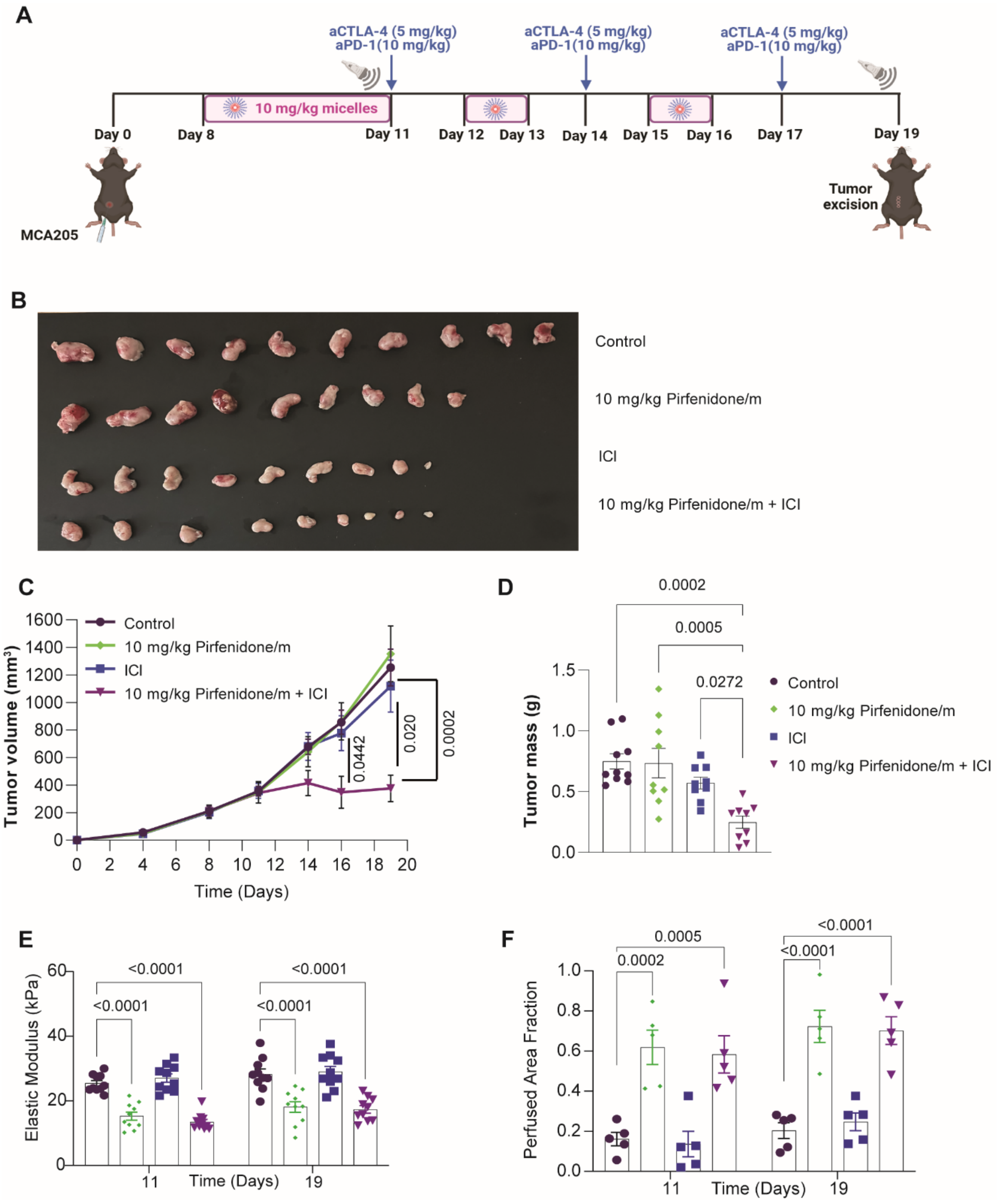

Supplementary Figure S10. **Pirfenidone micelles significantly improve the efficacy of immunotherapy in MCA205 tumors.** (A) Experimental treatment protocol. Created with BioRender.com. (B) Images of tumors after removal. (C) Tumor growth (n=10 mice), (D) tumor mass (n=10 mice), (E) tumor elastic modulus (n=5 mice, N=2 image fields per mouse)

measured with SWE and (F) Perfused area (n=5 mice) measured with CEUS of MCA205 tumors treated with Pirfenidone/m and anti-CTLA4/anti-PD-1 antibodies. Data are presented as mean ± SE. Statistical analyses were performed by using for (C) mixed-effects analysis with multiple comparisons Tukey test, for (D) ordinary one-way ANOVA with multiple comparisons Dunnett test and for (E, F) using two-way ANOVA with multiple comparisons Dunnett test.

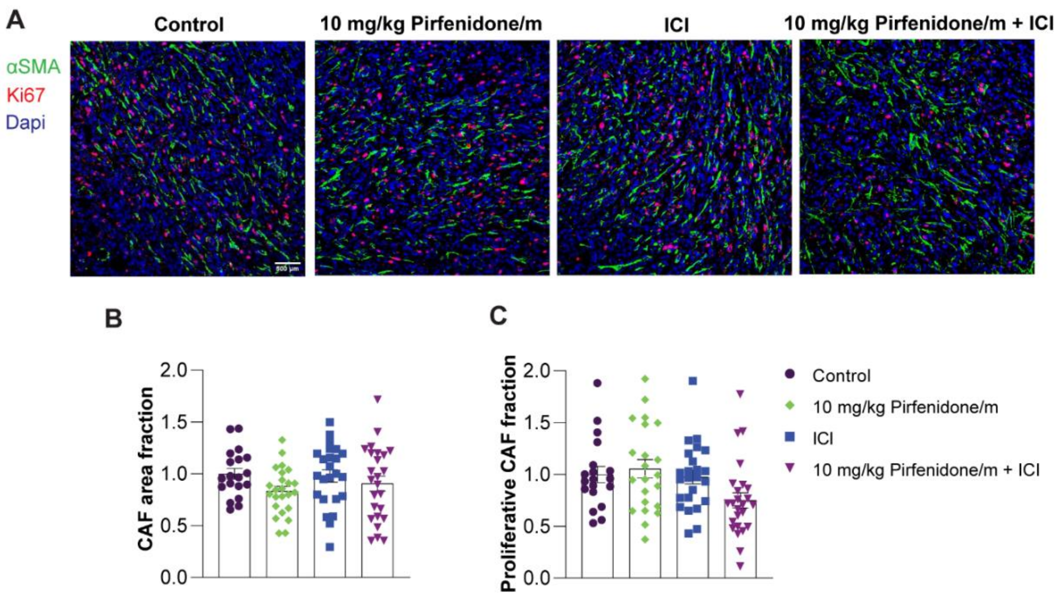

Supplementary Figure S11. **Effect of Pirfenidone micelles and Immunotherapy on CAFs activity in 4T1 tumors.** (A) Representative immunofluorescence images of CAF marker anti-αSMA (green) and Ki67 staining (red) counterstained with nuclear staining (blue). White scale bar indicates 0.5 mm. (B) Quantification of αSMA positive fraction normalised to DAPI nuclear stain. (C) Quantification of area double positive for αSMA and Ki67 marker as a measure of CAF proliferation normalized to total αSMA positive staining. Data are presented as mean ± SE. Statistical analyses were performed by using ordinary one-way ANOVA with multiple comparisons Dunnett test. P-values less than 0.05 are denoted on the graphs.

T cell gating

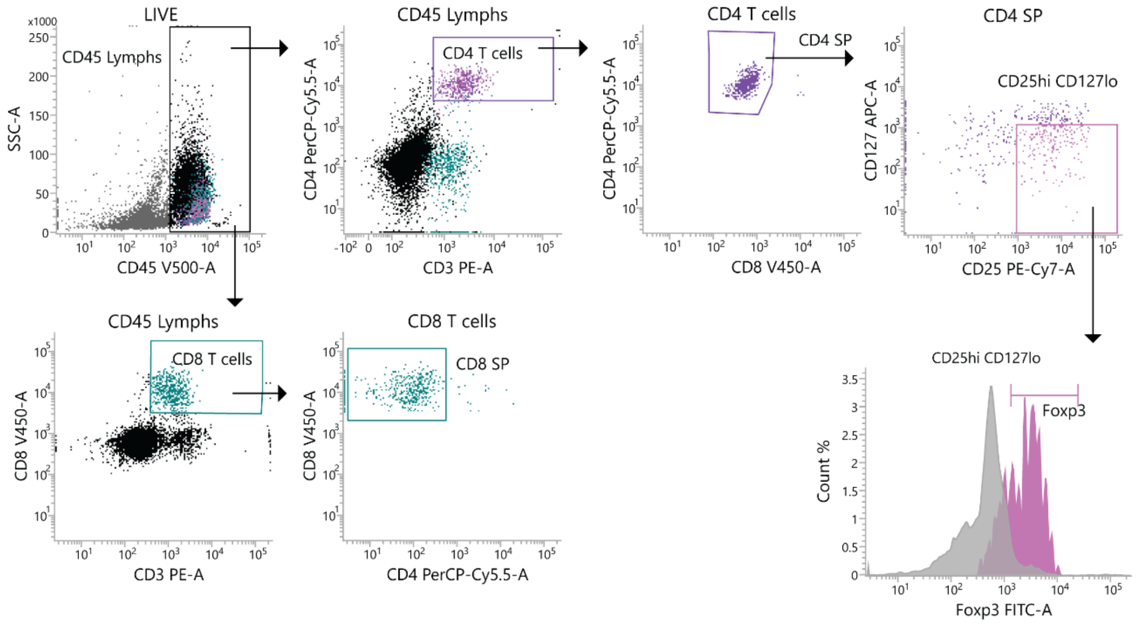

Macrophage gating

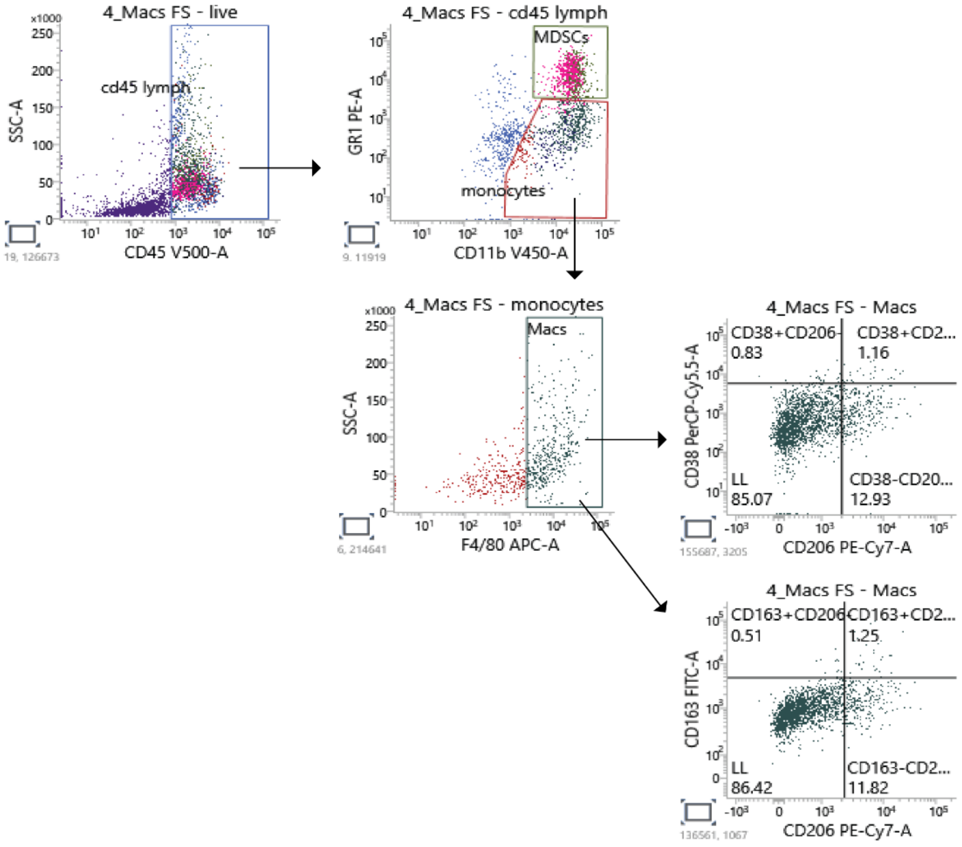

Supplementary Figure S12. Gating strategy with representative histograms of flow data corresponding to T cell and macrophage immunostaining.

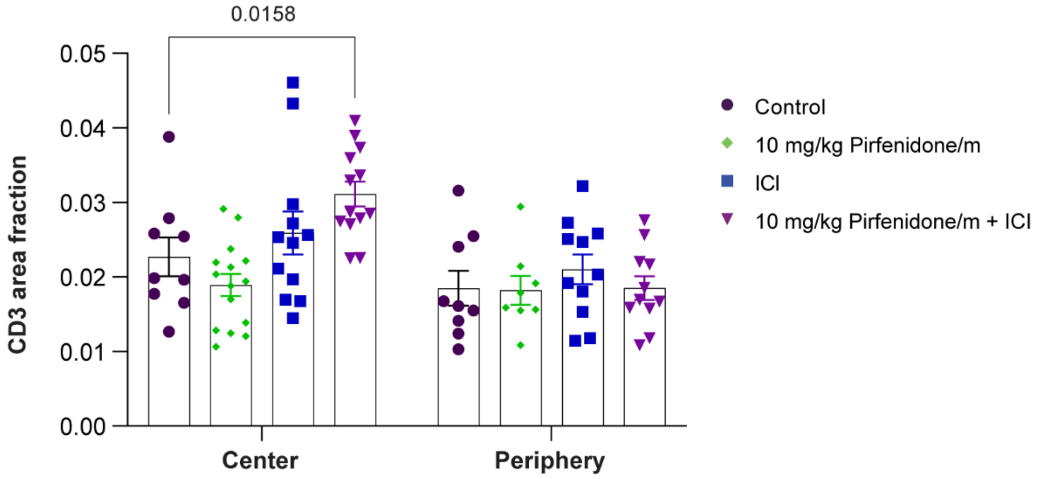

Supplementary Figure S13. **Pirfenidone micelles combined with Immunotherapy significantly increased CD3 T cell density in the center of the tumor.** Graph of the area fraction of the CD3 T cell marker in immunofluorescence images normalized to DAPI stain in the center and the periphery in 4T1 tumors.

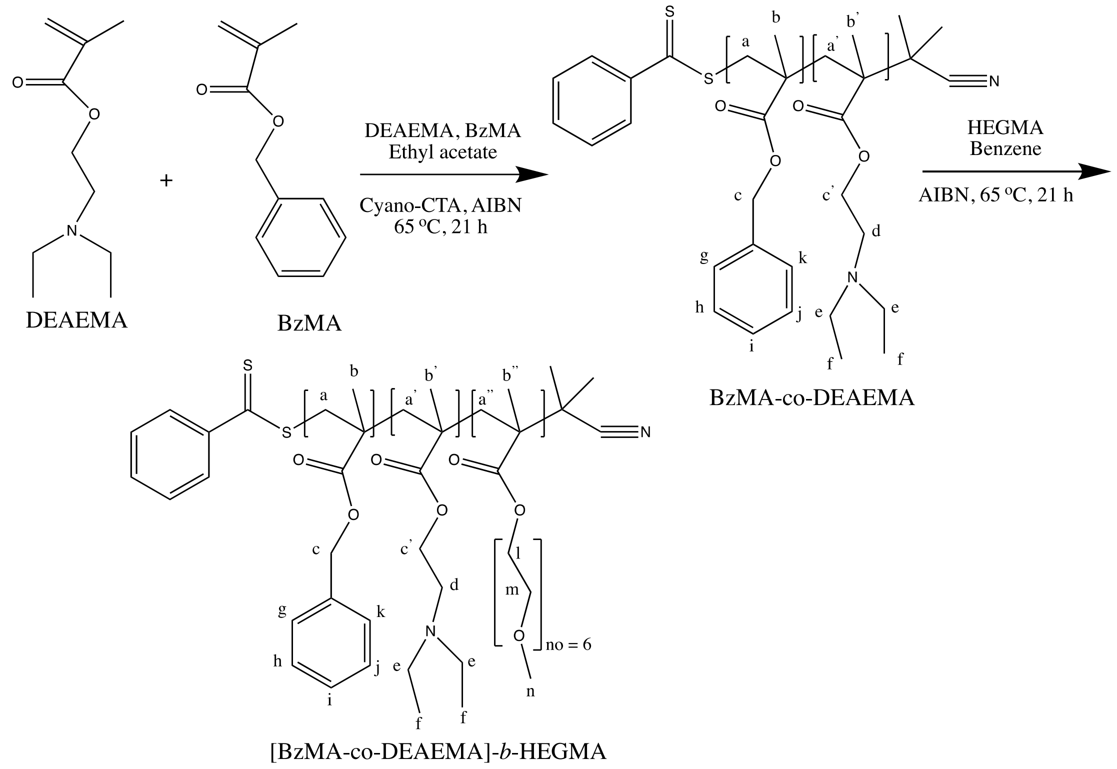

Supplementary Figure S14. Synthetic scheme followed for the preparation of the [$BzMA_{36}$-co-$DEAEMA_{6}$]-*b*-$HEGMA_{89}$ diblock copolymers by RAFT.

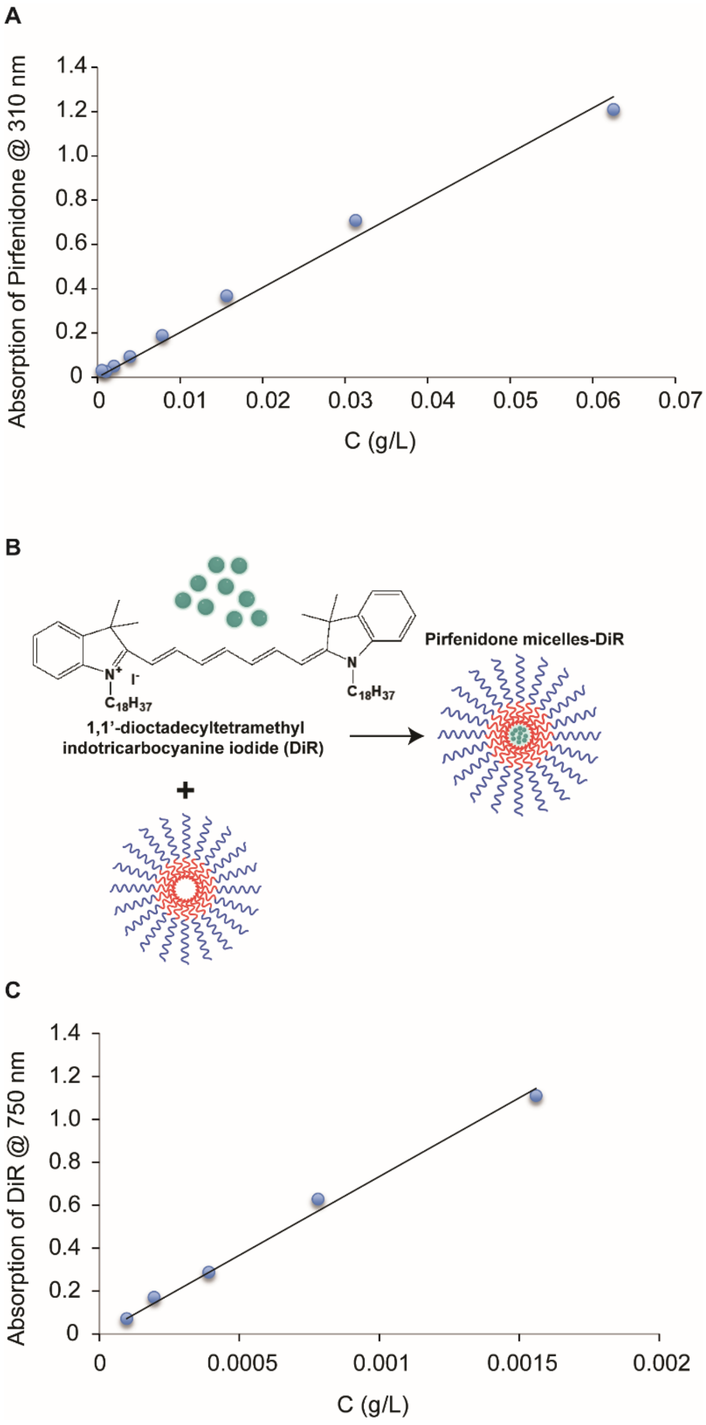

Supplementary Figure S15. (A) Absorption (at λ_{max} 310 nm) *versus* concentration calibration curve of Pirfenidone recorded in methanol. (B) Schematic of DIR Pirfenidone/m preparation. (C) Absorption (at λ_{max} 750 nm) *versus* concentration calibration curve of DiR recorded in acetone.

References

1. Stylianopoulos T, Martin JD, Chauhan VP, Jain SR, Diop-Frimpong B, Bardeesy N, Smith BL, Ferrone CR, Hornicek FJ, Boucher Y, Munn LL, Jain RK. Causes, consequences, and remedies for growth-induced solid stress in murine and human tumors. *Proceedings of the National Academy of Sciences of the United States of America* **2012**, *109*, 15101-15108.

2. Mpekris F, Voutouri C, Panagi M, Baish JW, Jain RK, Stylianopoulos T. Normalizing tumor microenvironment with nanomedicine and metronomic therapy to improve immunotherapy. *Journal of controlled release : official journal of the Controlled Release Society* **2022**, *345*, 190-199.

3. Averkiou M, Keravnou CP, Izamis ML, Leen E. Evaluation of Perfusion Quantification Methods with Ultrasound Contrast Agents in a Machine-Perfused Pig Liver. *Ultraschall in der Medizin* **2018**, *39*, 69-79.

4. Dietrich CF, Averkiou MA, Correas JM, Lassau N, Leen E, Piscaglia F. An EFSUMB introduction into Dynamic Contrast-Enhanced Ultrasound (DCE-US) for quantification of tumour perfusion. *Ultraschall in der Medizin* **2012**, *33*, 344-351.

5. Xin L, Yan Z, Zhang X, Zang Y, Ding Z, Xue H, Zhao, C. Parameters for Contrast-Enhanced Ultrasound (CEUS) of Enlarged Superficial Lymph Nodes for the Evaluation of Therapeutic Response in Lymphoma: A Preliminary Study. *Medical science monitor : international medical journal of experimental and clinical research* **2017**, *23*, 5430-5438.
